# Temperature Stress Induces Shift From Co-Existence to Competition for Organic Carbon in Microalgae-Bacterial Photobioreactor Community – Enabling Continuous Production of Microalgal Biomass

**DOI:** 10.3389/fmicb.2021.607601

**Published:** 2021-02-11

**Authors:** Eva Sörenson, Eric Capo, Hanna Farnelid, Elin Lindehoff, Catherine Legrand

**Affiliations:** ^1^ Department of Biology and Environmental Science, Centre of Ecology and Evolution and Microbial Model Systems, Linnaeus University, Kalmar, Sweden; ^2^ Department of Chemistry, Umeå University, Umeå, Sweden

**Keywords:** microalgae, bacteria, community, resilience, coexistence, competition, adaptive cycles, interactions

## Abstract

To better predict the consequences of environmental change on aquatic microbial ecosystems it is important to understand what enables community resilience. The mechanisms by which a microbial community maintain its overall function, for example, the cycling of carbon, when exposed to a stressor, can be explored by considering three concepts: biotic interactions, functional adaptations, and community structure. Interactions between species are traditionally considered as, e.g., mutualistic, parasitic, or neutral but are here broadly defined as either coexistence or competition, while functions relate to their metabolism (e.g., autotrophy or heterotrophy) and roles in ecosystem functioning (e.g., oxygen production, organic matter degradation). The term structure here align with species richness and diversity, where a more diverse community is though to exhibit a broader functional capacity than a less diverse community. These concepts have here been combined with ecological theories commonly used in resilience studies, i.e., adaptive cycles, panarchy, and cross-scale resilience, that describe how the status and behavior at one trophic level impact that of surrounding levels. This allows us to explore the resilience of a marine microbial community, cultivated in an outdoor photobioreactor, when exposed to a naturally occurring seasonal stress. The culture was monitored for 6weeks during which it was exposed to two different temperature regimes (21 ± 2 and 11 ± 1°C). Samples were taken for metatranscriptomic analysis, in order to assess the regulation of carbon uptake and utilization, and for amplicon (18S and 16S rRNA gene) sequencing, to characterize the community structure of both autotrophs (dominated by the green microalgae *Mychonastes*) and heterotrophs (associated bacterioplankton). Differential gene expression analyses suggested that community function at warm temperatures was based on concomitant utilization of inorganic and organic carbon assigned to autotrophs and heterotrophs, while at colder temperatures, the uptake of organic carbon was performed primarily by autotrophs. Upon the shift from high to low temperature, community interactions shifted from coexistence to competition for organic carbon. Network analysis indicated that the community structure showed opposite trends for autotrophs and heterotrophs in having either high or low diversity. Despite an abrupt change of temperature, the microbial community as a whole responded in a way that maintained the overall level of diversity and function within and across autotrophic and heterotrophic levels. This is in line with cross-scale resilience theory describing how ecosystems may balance functional overlaps within and functional redundancy between levels in order to be resilient to environmental change (such as temperature).

## Introduction

Microorganisms make up ≈70% of the aquatic biomass and their interactions in the microbial loop are vital for the recycling of energy and nutrients that ensure the ecosystem services provided by aquatic food webs (Azam et al., 1983; Bar-On et al., 2018). In addition, aquatic microorganisms contribute ≈50% of the O_2_ in the atmosphere today (Field et al., 1998; Behrenfeld et al., 2001). The impact of current and predicted environmental changes on aquatic microorganisms, including the increasing sea surface temperatures (Collins and Knutti, 2013), is difficult to assess due to the lack of studies using high-resolution molecular methods of microbial community interactions. The ability of aquatic microbial ecosystems to be resilient to disturbances, on shorter or longer scales, depends on the interplay of multiple factors (Allison and Martiny, 2008; Shade et al., 2012a). Identifying the behavior of key resilience mechanisms in response to changed environmental conditions may lead to more accurate predictions of the effects of environmental changes on biogeochemical cycles. Such knowledge could for instance enable the implementation of more locally adapted monitoring and management programs of aquatic microbial ecosystems (Bernhardt and Leslie, 2013; Andersson et al., 2015). Several studies have suggested that the functional capabilities of experimental microbial ecosystems, and thus their resilience, were not found to be related to the composition of the communities (Fernandez et al., 2000; Wang et al., 2011; Vanwonterghem et al., 2014; Louca and Doebeli, 2016), which might be explained by the large functional redundancy and diversity that exists among microbial species (Louca et al., 2017, 2018, 2020). Microbial ecosystems are complex and consist of several interacting levels, such as trophic levels, that enable the transfer of energy and nutrients within the microbial loop and further up in the food web. Adaptations to changed conditions seen at one level likely have an influence on the levels above or below (Gunderson and Holling, 2002). Thus, in order to gain a deeper understanding of the underlying mechanisms of the resilience of microbial communities, it is important to link experimental results with theories. In this study, we focused on three interlinked mechanisms that together have the potential to influence microbial ecosystem resilience in response to changed environmental conditions: biotic interactions, functional adaptations, and community structure. Interactions between organisms in microbial ecosystems are commonly described through the presence or absence of the exchange of signals or metabolites, including mutualistic, parasitic, or neutral relationships (Tipton et al., 2019). Here, the focus is on broad-scale community interactions, disregarding any potential microalgal-bacterial cooperation apart from that that involves carbon. Broadly, the considered interactions may primarily be characterized by either coexistence, governed by resource partitioning (Sörenson et al., 2020), or by competition for energy and nutrients, which may lead to competitive exclusion (Schoener, 1974; Chesson, 2000). Both types of interactions influence biogeochemical cycles, e.g., that of carbon, through potential functional changes and variations in microbial community structure (Lindh and Pinhassi, 2018; Sörenson et al., 2020). Functions relevant for studies of aquatic microbial ecosystems commonly relate to whether organisms are autotrophs, heterotrophs, or mixotrophs, which is defined by the type of carbon (inorganic, organic, or both) they have the capacity to acquire as a food source and to use for energy production. Temporal dynamics in the structure of a community relate to its species richness or diversity, in which a more diverse community is characterized by a more efficient use of resources compared to a less diverse community that likely have a more narrow functional range (Cardinale et al., 2006; Ptacnik et al., 2008).

In resilience theory, the term panarchy has been used together with adaptive cycles and cross-scale resilience theories to describe the sustainability of both social and ecological systems (Holling, 1973; Peterson et al., 1998; Gunderson and Holling, 2002). Adaptive cycles postulate four phases that a system continuously pass through: birth – growth and accumulation of resources (*r*), maturation – conservation of established processes (*K*), death – the release upon changed conditions (*Ω*), and renewal – the creative phase of reorganization and adaptation to new conditions (*α*; Holling, 1973). Panarchy describes how separate levels within an ecosystem, each with their own adaptive cycle, interact in order to accommodate and adapt to changed conditions. Where lower levels, primarily when entering the Ω-phase, influence the level above (termed revolt) while the upper levels, primarily during the K-phase, are able to buffer the impact (termed remember), and thereby the levels together affect the community resilience (Gunderson and Holling, 2002). Cross-scale resilience describes how ecosystems may become resilient by balancing overlapping functional diversity within and functional redundancy across levels (Peterson et al., 1998). In this study, levels are interpreted as trophic levels.

Ecosystem resilience may be explained as the capacity to harbor, through internal fluctuations of function and structure, smaller or larger environmental changes (Holling, 1973), while maintaining over-all function, structure, and identity (Walker, 2004). The capacity of aquatic microbial ecosystems to respond in a resilient manner to the regime shifts in, e.g., temperature that might be the result of present and future climate change is difficult, by important, to assess (O’Gorman et al., 2012). Currently, few studies have empirically investigated resilience within aquatic microbial ecosystems (e.g., Shade et al., 2012b; Lindh and Pinhassi, 2018). For the coastal regions of Scandinavia projected environmental changes are increasing temperature, precipitation, land run-off, and ocean acidification (Collins and Knutti, 2013). Coupling analyses of the responses in controlled and simplified ecosystems to environmental change, in terms of structural and functional dynamics together with analyses of the impact on community interactions, with established ecological theories, models of aquatic ecosystem responses to climate change may be improved (Prosser and Martiny, 2020).

Using model systems with only a few species and controlled conditions in a laboratory help to gain a regulatory mechanistic insight of microbial interactions at the detailed level (Segev et al., 2016; Bolch et al., 2017). It is, however, important to study more complex, yet simplified systems, with several interacting levels, as ecosystem responses to environmental change, depend on the response at each contained level (Gunderson and Holling, 2002). Thus, systems of medium complexity, with several interacting functional groups (auto-, hetero-, and mixotrophs), kept under controlled nutrient conditions and influenced by a few environmental parameters, will help in predicting the consequences of environmental change on microbial communities and the impact of this on larger scale biogeochemical cycles (Otwell et al., 2018). In the present study, an algal polyculture kept in an outdoor photobioreactor (PBR), with a capacity to produce up to 0.88gl^−1^ biomass per day (Supplementary Figure S1), was investigated. The PBR community, composed of a few naturally selected microalgae species, dominated by a mixotrophic green microalgae (with the ability to utilize both inorganic and organic carbon), and a mixed, naturally established, bacterial community, was provided with inorganic carbon, and studied under two different temperature conditions (warm/cold). As the availability of light influence the efficiency of photosynthesis and uptake of carbon, this was also studied in addition to temperature as a potential structuring factor. The aim of the study was to elucidate the effect that changes in temperature regimes have on microalgae-bacteria interactions, by focusing on the functional regulation in the acquisition of carbon (organic and/or inorganic) and on the impact of this regulation on the dynamics of community structure. Further, we wanted to investigate the influence of interlevel interactions on the resilience of the community, in terms of maintained production of microalgal biomass. Analyses of community structural dynamics were made by generating amplicon sequencing data and using co-occurrence network analysis. Analyses of the functional regulation in the acquisition of carbon by the PBR community were made using a metatranscriptomic approach. The capacity of the microbial community for resilience was investigated using adaptive cycles, panarchy, and cross-scale resilience theories.

## Materials and Methods

### Photobioreactor Setup

**Figure 1:**
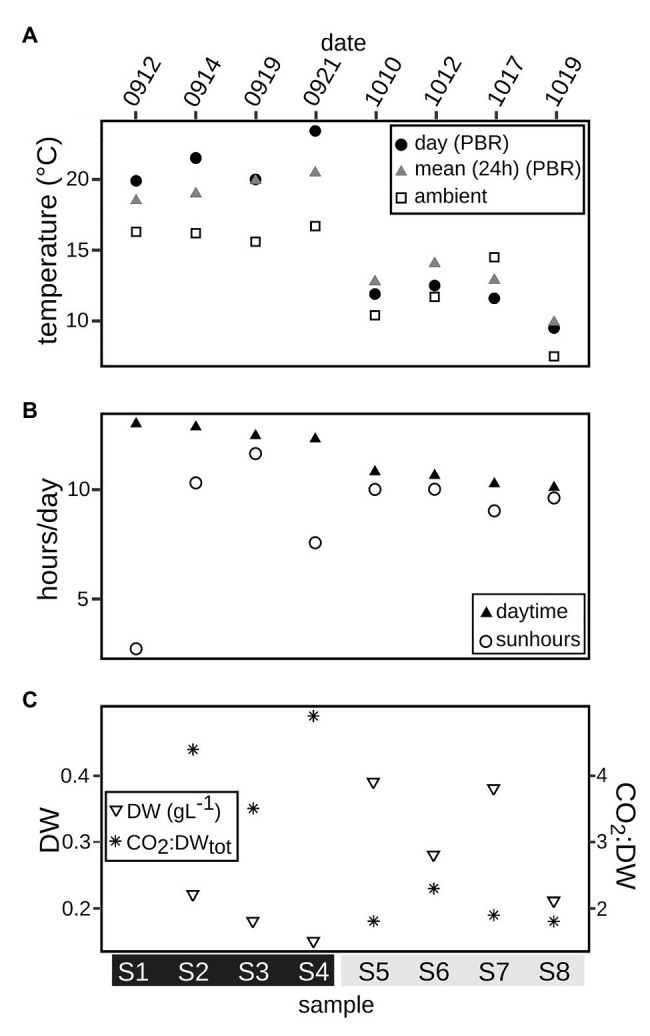
Parameters associated with the photobioreactor (PBR) during the sampling period, dates of sampling specified on top, sample names S1–S8 at the bottom. **(A)** Daytime temperature (°C) in PBR (closed circles), mean temperature per 24h in PBR (gray triangles), regional ambient daytime temperature (measured by SMHI; open squares). **(B)** Number of hours day^-1^ with sunshine (direct solar radiation > 120Wm^–2^, 10km south of PBR location; open circles), daytime hours/day (black triangles). **(C)** For S2–S8 sampling events (S1 omitted due to lack of data), left *y*-axis: Dry weight (DW) of biomass (gl^−1^; open triangles) and right *y*-axis: ratio of flow in added mass (g) of CO_2_:total dry weight (in 3200L; asterisks).

A large-scale outdoor PBR, PBR, remediating cement factory flue gas emissions between April and November since 2014 (Olofsson, 2015; Mattsson et al., in prep), located at the southern part of Öland (56°21.2'N 16°24.6'E, Sweden), in the Kalmar Strait/Baltic Proper, was sampled bi-weekly (Wednesdays and Fridays), around 10a.m., during September and October of 2018. The closed non-heated system containing 3,200L brackish (salinity 6.9 ± 0.3) Baltic Sea water, was circulated between eight vertical flat panels and designed to take up CO_2_ from the emitted flue gas through algal photosynthesis. Stable pH (7.8 ± 0.3) and O_2_ levels were monitored and maintained within a constant range. After each bi-weekly sampling (1L) and subsequent harvest of biomass (30–50% of total volume), the reactor was supplied with nutrients in the form of Cell-Hi f/2 powder (Varicon Aqua), according to Guillard’s f/2 medium (Guillard, 1975), amended with 19mM of NaH_2_PO_4_, along with a refill of filtered seawater (0.2μm cylinder polypropylene filters cartridge), to the full volume. The 6weeks covered in the study included two periods, with four sampling events each: S1–S4 and S5–S8 respectively, with distinct seasonally induced temperature conditions (Figure 1). The light reaching the panels was reduced by thin nets with a 40–60% reduction efficiency, with negligible influence on reactor panel temperature, between first and second sampling occasion each week (corresponding to sampling events S4, S6, and S8, Figure 1).

### Measurements of Biomass and Environmental Parameters

Algal biomass was measured as dry weight (DW) of 10ml algal culture for each date. The culture was filtered onto rinsed, pre-dried and weighed, 47mm GF/F filters (0.5μm, Whatman), and dried again at 100°C overnight. The weight of the dry algal biomass was measured the following day. Daily sunlight hours were measured using a CSD three sunshine duration sensor (Kipp & Zonen), counting direct solar radiation >120Wm^2–1^. The sensor was located at Ottenby (ca 10km south of the location of algal PBR). Daytime length was retrieved from Soltimmar.se (location Kalmar). Temperature in the PBR panels was measured by the monitoring system of the reactor. Ambient temperature of the region was retrieved from the Swedish Meteorological and Hydrological Institute (SMHI).

### 16S and 18S Amplicon Sequencing Analysis

For DNA sampling, 5–7ml of culture was filtered, with a vacuum hand-pump, through 0.2μm pore-sized filters (Supor-200, 47mm, PALL), with three replicates per sampling event. All filters were immediately immersed in RNAlater (Sigma Scientific) and snap-frozen in dry ice, and upon return to the laboratory (~1–2h later) stored at −80°C, until further processing.

DNA was extracted using the FastDNA Spin Kit for soil (MPBio, Irvine, CA, United States) according to protocol, with the modification that a 1h incubation step at 55°C with proteinaseK (20mg/ml final concentration) was included after the homogenization step, to increase DNA precipitation. The 48 DNA extracts were quantified using a Qubit fluorometer (Invitrogen, Carlsbad, CA, United States) and checked for purity with Nanodrop 2000 spectrophotometer (Thermo Fisher Scientific, Waltham, MA, United States). The V3–V4 region of the 16S rRNA gene was amplified using primers 341F (CCTACGGGNGGCWGCAG) and 805R (GACTACHVGGGTATCTAATCC), and the V4–V5-region of the 18S rRNA gene was amplified using primers 574*F (CGGTAAYTCCAGCTCYV) and 1132R (CCGTCAATTHCTTYAART), connected to Nextera DNA Dual-index adaptors in accordance with Herlemann et al. (2011) and Hugerth et al. (2014), respectively. The PCRs were performed using Phusion Mastermix (Thermo Scientific), and the following settings: (i) 16S [1 x (98°C, 30s), 20 x (98°C, 10s, 58°C, 30s, 72°C, 15s), 1 x (72°C, 2min)], modified from Bunse et al. (2016), (ii) 18S [1 x (98°C, 30s), 28 x (98°C, 20s, 50.4°C, 20s, 72°C, 15s), 1 x (72°C, 2min)]. In a second PCR assay, Nextera indices (i7 and i5) were attached to both the 16S and 18S products with settings: [1 x (98°C, 30s), 12 x (98°C, 20s, 62°C, 30s, 72°C, 30s), 1 x (72°C, 2min)], modified from Hugerth et al. (2014). The PCR products were purified both after the first and the second amplification steps, using the Agencourt AMPure XP kit (Beckman-Coulter) according to the manufacturer’s instructions. Products were quantified using a Qubit fluorometer and quality checked using a NanoDrop 2000 spectrophotometer. Fragment sizes were validated to ca 600 base pairs (bp) for both 16S and 18S samples using gel electrophoresis. Samples were pooled at equimolar concentrations, and the pool was purified using E.Z.N.A Gel extraction Kit (Omega Bio-tek) and sequenced using Illumina MiSeq v3, PE (Illumina Inc., United States), 2 × 300bp, at SciLifeLab (Stockholm, Sweden).

Raw reads of 16S and 18S rRNA gene amplicon data were processed separately with dada2 (version 1.6.0; Callahan et al., 2016), implemented in R (version 3.4.3; Core, 2018). Of the 6,434,529 18S raw reads, 76% remained after error model filtration, and of the 12,981,918 16S reads, 87% remained after error model filtration (Supplementary Tables S1, S2). Only forward reads were used to construct the sequence table of amplicon sequence variants (ASV’s), due to uninformative overlaps of reverse reads. This resulted in 1,110 18S ASVs and 6,467 16S ASVs, which were used for further analyses. For both 16S and 18S ASVs the taxonomy was assigned using SILVA database (v132; Quast et al., 2013). Of the 16S ASVs 299 were assigned as chloroplasts (Supplementary Figure S2), which were filtered from the 16S dataset before further analyses. Relative abundances of both 16S and 18S ASVs were plotted in R with ggplot2 (Wickham, 2016), and an assessment of independent environmental parameters was made using function *varclus* from R package Hmisc (Harrell and Dupont, 2019) with Spearman’s rank correlations. Canonical correspondence analysis (CCA) was made with independent environmental parameters in the model, with function *cca* (999 permutations) from R package vegan (Oksanen et al., 2008) and plotted with ggplot2. PERMANOVA analyses of both 16S and 18S ASVs were made using *adonis2*, richness (Chao1) and diversity (Shannon and Simpson) were estimated using functions *estimateR* and *diversity*, respectively, from the vegan package (Oksanen et al., 2008). For the 16S data, functions *rarefy* and *rarecurve*, with step = 20, from the vegan package were used to make rarefaction curves, and R package RAD analysis (Saeedghalati et al., 2017) was used to make normalized, by minimum richness = 136, rank abundance curves. As most environmental parameters were missing from the initial date (0912, S1) this date was excluded from the CCA’s.

Amplicon data was analyzed for patterns of co-occurrences between relative abundances of 18S and 16S ASVs with environmental parameters including temperature, light/shade, sun hours, and biomass (DW), using the R package Weighted Correlation Network Analysis (WGCNA, v1.68) (Langfelder and Horvath, 2008) in R (v3.6.1). The 18S and 16S datasets were first rarefied to the smallest library size (39,469 and 10,824 sequences, respectively) and to reduce the complexity of the data, ASVs with <0.1% counts per library were excluded, resulting in 55 18S ASVs and 309 16S ASVs that were used for further analysis. Functions from WGCNA R package was performed according to Capo et al. (2017). Briefly, relative abundance data was standardized with Hellinger transformation (function *decostand*; Oksanen et al., 2019). A signed network of clustered ASVs was created using function *adjacency* and a minimum of eight nodes (ASVs) per module, and power four was used as the threshold value. The relationships between the values of environmental factors, and modules eigenvalues were displayed using a heatmap. Only edges with pair-wise correlations values >0.3 and positive Pearson coefficient correlation’s values >0.34, were included for network visualization, made using the software Gephi (Bastian et al., 2009).

### Metatranscriptomic Analysis

For RNA sampling, 7.5–10ml of culture was filtered sequentially, using a vacuum hand-pump, through 3μm pore-sized filters (Versapor-3000, 47mm, PALL) followed by 0.2μm pore-sized filters (Supor-200, 47mm, PALL), with six replicates per sampling event. All filters were immediately immersed in RNAlater (Sigma Scientific) and snap-frozen in dry ice, until −80°C conditions were available (~1–2h later).

Within 4months after sampling, filters were thawed on ice. 12 filters (6 × 0.2 and 6 × 3μm) were retrieved per sampling occasion, of which one filter each of 0.2 and 3μm were combined, resulting in six replicates. Filters were combined for RNA-extraction to maximize yield. On ice, filters were cut with scissors, and placed in MatrixE tubes (MPBio) prepared with, in total 1ml, of RLT-buffer (Qiagen, Venlo, Netherlands), TE-buffer, B-mercaptoethanol [1:100], and Lysozyme (0.04mg/ml). A FastPrep-24 instrument with a QuickPrep adaptor (MPBio) was used for lysing the cells, three rounds each at 6ms^−1^ for 40s, with 1min on ice in between runs. After lysing, RNA was extracted using Qiagen RNeasy mini kit, according to protocol. The extracted RNA was treated with DNase to remove DNA (AMBIONTurbo DNA free). At this stage 24 samples were sent for poly-A selection followed by mRNA fragmentation and synthesis of cDNA at SciLifeLab (Stockholm, Sweden), to be used for eukaryotic gene expression analyses. Remaining 24 samples, to be used for prokaryotic gene expression analyses, were depleted of rRNA using RiboMinus Transcription isolation kit (Invitrogen), with a RiboMinus Concentration module. This was followed by a cDNA to aRNA protocol (MessageAmp II-Bacteria RNA amplification kit, Invitrogen). All 48 samples were sequenced on one lane of Illumina NovaSeq 6000 S1, PE 2 × 150 bp, at SciLife lab in Stockholm, Sweden.

Metatranscriptomes obtained from the poly-A selected (eukaryotic) and the amplified aRNA (prokaryotic) fractions were computationally processed using the same procedure. Quality was initially checked with FastQC (v0.11.8; Andrews, 2009) and MultiQC (v1.7; Ewels et al., 2016). Adaptors were removed using Cutadapt (v2.3; Martin, 2011). An additional check with FastQC/MultiQC showed that the remaining adaptors were below 0.1% for all samples. Reads were quality trimmed using Sickle (v1.33; Joshi and Fass, 2011). For the eukaryote data, there were in total 1,019,068,864 raw reads of which 94 ± 1% remained after quality filtration. For the prokaryote data, there were in total 966,439,508 raw reads of which 99 ± 0.4% remained after quality filtration (Supplementary Tables S3, S2). rRNA was filtered out by aligning reads to a local rRNA database using Bowtie2 (v2.3.5.1; Langmead and Salzberg, 2012). Samtools (v 1.9; Li et al., 2009) was then used to retrieve reads that did not match to the rRNA-db. Reads were assembled using Megahit (v.1.1.2; Li et al., 2016), and annotated against NCBI-RefSeq protein db, using Diamond (v0.9.24; Buchfink et al., 2015). These resulted in 571,442 eukaryote open reading frames (ORFs) and 58,151 prokaryote ORFs, of which 149,303 (26%) and 38,113 (66%) ORFs, respectively, were functionally annotated with SEED db (July 2019) using MEGAN (community edition, v6.12.8; Huson et al., 2016). MEGAN was also used for taxonomic annotations to NCBI-nr db (July 2019). Reads were then mapped against the assembly with Bowtie2 (v2.3.5.1) and Samtools (v 1.9).

### Data Deposition

Sequence data have been submitted to European Nucleotide Archive, ENA, understudy ERP116148; amplicon raw reads: ERR3419055-ERR3419102; metatranscriptome raw reads: ERR3421213-ERR3421260.

## Results

### Temporal Changes in Temperature and Light Conditions During the Experiment

The PBR was located outside throughout the study at northern hemisphere fall conditions (Figure 1). During the initial sampling period (S1–S4), the average daytime temperature in the PBR was significantly higher, at an average temperature of 21 ± 2°C (average ambient temperature 16 ± 0.5°C), compared to the second period (*t*-test, *p* = 3.1e-14), starting 2weeks later (S5–S8), at an average temperature of 11 ± 1°C (average ambient temperature 11 ± 3°C). Between the first sampling (S1) and the last (S8), the length of daytime decreased by 2h and 55min. Daily sunshine hours varied between 2 and 11h during the warmer period, while it was more stable, 9–10h, during the colder period (Figure 1).

### Nutrient Conditions and Production of Biomass

Inorganic nutrients (NH_4_ and PO_4_) were added to the PBR to assure non-limiting concentrations during the study period (Supporting Information Table 1). Significantly less biomass was produced during the initial (warmer) period (0.18 ± 0.04gl^−1^) compared to the second (colder) period (0.32 ± 0.09 gl^−1^; *t*-test, *p* = 7.8e-05; Figure 1). The increased production pushed the capacity of the system towards carbon limitation, shown by the ratio between the flow of CO_2_ to total biomass (gl^−1^) produced, that shifted from 3.5 to 4.9 during the warmer period to 1.8–2.3 during the colder period (Figure 1).

### Environmental Parameters Structuring the PBR Microbial Community

**Figure 2:**
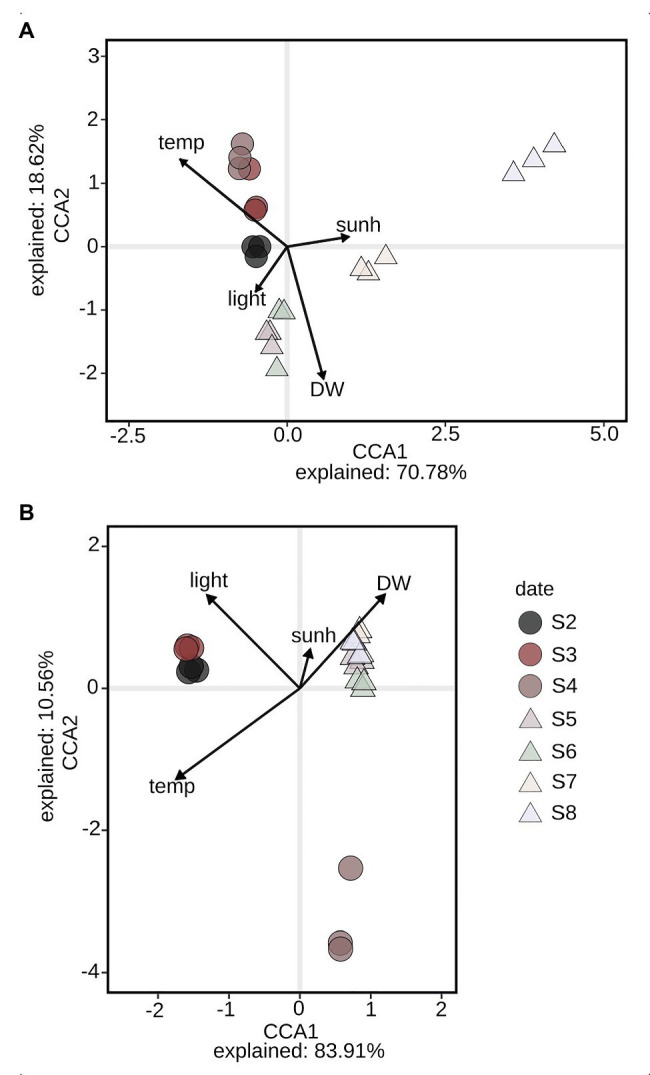
Canonical correspondence analysis (CCA) biplot of the eukaryote **(A)** and prokaryote **(B)** community, plotting samples with independent environmental parameters at each date of sampling (S2–S8) in the reactor: Temp – temperature in PBR (°C), DW – biomass dry weight (gl^−1^), sunh – number of sunshine hours at day of sampling (h), light – unshaded/shaded (reduced light input by 40–60%). Dates are denoted by color, warm period by (○), and cold period by (△). Date S1 is excluded due to missing nutrient data.

Analyses of the amplicon sequencing data with CCA (Figure 2A) and PERMANOVA showed that the structure of the microbial eukaryotes was significantly affected by the temperature in the PBR (PERMANOVA; temp, *df* = 2; *F* = 11.8, *R2* = 0.34, *p* = 0.004**) but not by the availability of light (reduced by shading; PERMANOVA; light, *df* = 2; *F* = 2.2, *R2* = 0.17, *p* = 0.069), while the structure of the microbial prokaryotes was significantly affected by both temperature and light (PERMANOVA; temp, *df* = 2; *F* = 27, *R2* = 0.55, *p* < 0.001***; light, *df* = 2; *F* = 8.0, *R2* = 0.43, *p* < 0.001***; Figure 2B). Further, the CCA plots suggested that the algal biomass, measured in DW (gl^−1^), was clearly linked to the structure of both the microbial eukaryotes and prokaryotes, and was highly dependent on the temperature regime (warm or cold; Figure 2).

### Composition of Microbial Eukaryotes

**Figure 3:**
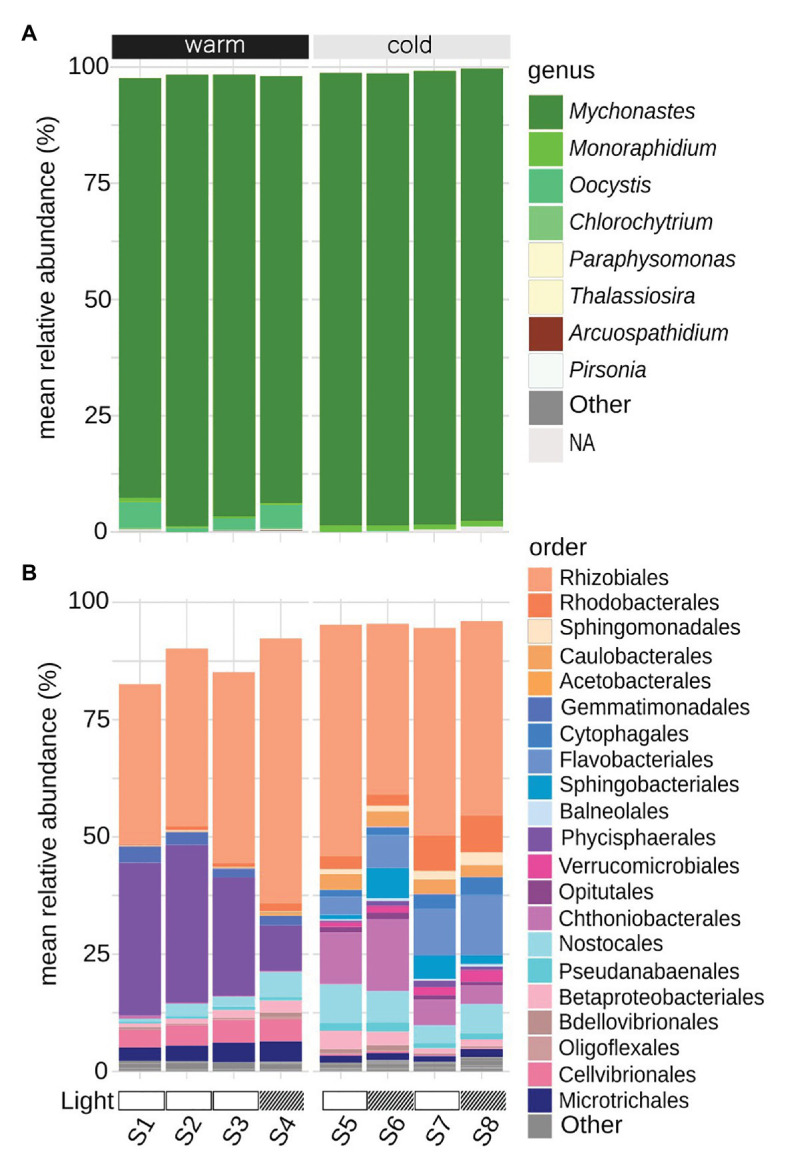
Taxonomic affiliation of ASVs, mean of triplicates per sampling date (S1–S8), with a relative abundance > 0.1%. **(A)** 18S ASVs with assigned taxonomy at the genus level. **(B)** 16S ASVs with assigned taxonomy at the order level. Warm: 19.5 ± 0.89°C, cold: 12.4 ± 1.76°C (mean temperature per 24 h in PBR); Light: light reduction, open bar → natural light, striped bar → light reduced by 40–60% by shading.

The 18S ASVs with a relative abundance >0.1% were taxonomically annotated at the genus level, corresponding to 98–99% relative abundance per sample (Figure 3A). The majority of sequences (ranging between 90 and 98% in samples) were annotated as green microalgae *Mychonastes*, of which one ASV (ASV_1) dominated all samples with relative abundances of 85–95% per sample (Supplementary Figure S3). The ASV had 100% sequence similarity to *Mychonastes* sp. with GenBank accession number MF595077 (Supplementary Figure S4). The average relative abundance of ASV_1 increased from the warm to the cold period. This coincided with a significant drop in both average species richness from 65 ± 11 to 38 ± 11 (std; Chao1; *t*-test, *p* = 4e-06) and average diversity from 0.65 ± 0.22 to 0.43 ± 0.09 (std; Shannon Index; *t*-test, *p* = 0.007; Supplementary Figure S5A). During the warm period, the relative abundance of ASVs assigned to green microalgae *Oocystis* (Supplementary Figures S3, S4) fluctuated between 1 and 6%, while they were almost absent during the colder period, as did the ASVs assigned to green microalgae *Chlorochytrium*, with relative abundances 0.1–0.4% during the warm period while they were not present during the cold period (Supplementary Figures S3, S4). Instead, ASVs assigned to the green microalgae *Monoraphidium* (Supplementary Figures S3, S4), increased from 0.3 to 0.8% during the warm period to a relative abundance of 1% during the cold period (Figure 3A).

### Composition of Microbial Prokaryotes

**Figure 4:**
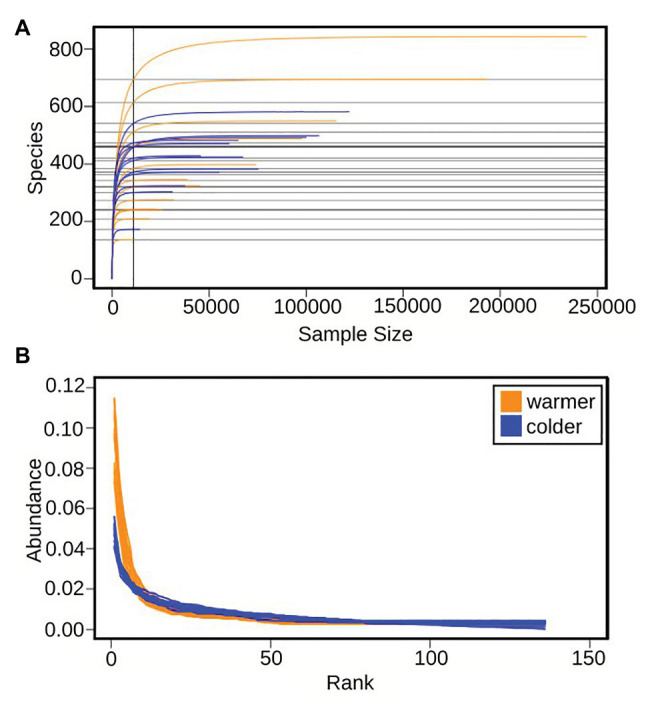
Diversity measures of 16S ASVs, excluding ASVs annotated as chloroplasts. **(A)** Rarefaction curves, plotting sample size vs. number of ASVs. **(B)** Normalized rank abundance curves, plotting ASVs, ranked by abundance vs. abundance (counts). Orange – warmer period, blue – colder period.

The 16S ASVs with a relative abundance >0.1%, excluding ASVs annotated as chloroplasts, were annotated at the order level (Figure 3B). Rarefaction curves indicate that the sample depth, including both warm and cold periods, were sufficient to capture the species richness within the community (Figure 4). There was a significant drop in species richness from an average of 492 ± 133 to 300 ± 113 (std) going from warm to cold temperature (*t*-test, *p* = 0.001; Supplementary Figure S5B) and normalized rank abundance curves suggest that rank abundances were lower for the colder period, compared to the warmer period (Figure 4). There was however a significant increase in average Shannon index diversity from 4.8 ± 0.1 to 5.1 ± 0.3 (std; *t*-test, *p* = 0.03), that take both abundance and evenness into account (Supplementary Figure S5B), indicating an increased diversity among the bacteria during the cold period. During the warm period, the bacterial community was dominated by alphaproteobacterial Rhizobiales (45 ± 9% relative abundance) and planctomycetal Phycisphaerales (26 ± 11% relative abundance), together making up >66% of the community (Figure 3B). Gammaproteobacterial Cellvibrionales, actinobacterial Microtrichales, and gemmatimonadetal Gemmatimonadales occurred during this period at low relative abundances 3–5%, and they were reduced to <1% of relative abundance during the colder period (Figure 3B). Several bacterial orders increased as the temperature dropped, verrucomicrobial Chthoniobacterales (9 ± 5% relative abundance), bacteriodetal Flavobacteriales (9 ± 4% relative abundance), and alphaproteobacterial Caulobacterales (at 3% relative abundance), while the occurrence of previously dominating order Phycisphaera was reduced to <1% relative abundance, as was less frequent planctomycetal Pirellulales (from 1% relative abundance to 0.8%). Rhizobiales remained at similar levels during the whole study (44 ± 7% relative abundance). Three orders increased, Rhodobacterales (from 2% relative abundance to 5 ± 3%), cyanobacterial Nostocales (from 4 ± 2% relative abundance to 7 ± 2%) and Betaproteobacteriales (from 2 ± 1% relative abundance to 3 ± 1%; Figure 3B).

### Differential Functional Gene Expression Analysis in the PBR Microbial Community

As the study covered two periods, capturing a temperature shift from warmer to colder temperature, raw unfiltered counts, normalized by contig length, of the metatranscriptome data were by differential expression analysis contrasted either for temperature regime or for the availability of light (affected by shading). This was done in order to establish which of the two factors influenced the functional repertoire the most. For the eukaryote data, the availability of light (shaded vs. not shaded) gave 53 (0.01%) significant (log2 fold changes, *p*adj < 0.01) differentially expressed ORFs, while temperature in the PBR (warm vs. cold) gave 114,783 (21%) significant (log2 fold changes, *p*adj < 0.01) differentially expressed ORFs. For the prokaryote data, 358 (0.6%) ORFs were significantly (log2 fold changes, *p*adj < 0.01) differentially expressed, when contrasted for the availability of light, while temperature resulted in 7438 (13%) significant (log2 fold changes, *p*adj < 0.01) differentially expressed ORFs. Temperature was thus identified as the most influential factor compared to light, for both eukaryotes and prokaryotes. The analysis of the gene expression data was focused on ORFs annotated to processes related to the acquisition and utilization of carbon and the log2 fold changes and adjusted *p*-values of included ORFs are given in Supplementary Tables S5 (eukaryotes) and S6 (prokaryotes).

### Eukaryotic Gene Expression Associated With Acquisition of Carbon

**Figure 5:**
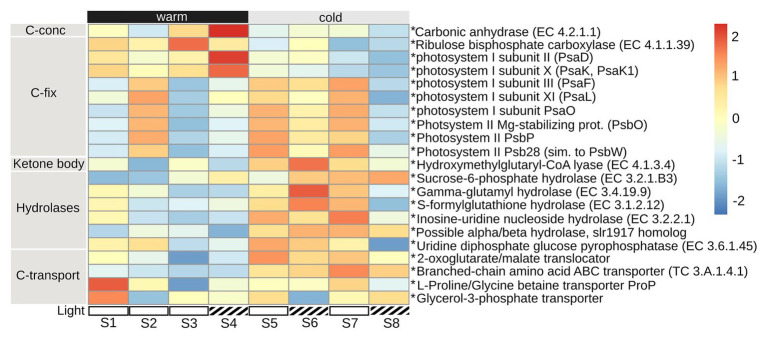
Eukaryote gene expression (metatranscriptome) of mechanisms associated with carbon acquisition. Included are key enzymes representative of: carbon concentration (C-conc) and carbon fixation (C-fix), ketone body formation, hydrolases, and carbon transport. Heatmap shows the mean of triplicates per sampling date (S1–S8), of counts normalized to TPM and then square root transformed. Heatmap was made using R package pheatmap, with setting scale by “row.” Warm: 19.5 ± 0.89°C, cold: 12.4 ± 1.76°C (mean temperature per 24 h in PBR); Light: light reduction, open bar → natural light, striped bar → light reduced by 40–60% by shading. * indicate *p*adj < 0.01 for log2 fold change of differential expression, contrasted for warm vs. cold time period.

Eukaryote metatranscriptomic data indicated expression of enzymes associated with photosynthesis (Ribulose bisphosphate carboxylase, Rubisco, EC 4.1.1.39) and the carbon concentrating enzyme carbonic anhydrase (EC 4.2.1.1), primarily during the period with warmer temperature (S1–S4), along with representatives from photosystem I (PsaD, PsaK, PsaK1; Figure 5). Other components of photosystem I (PsaF, PsaL, PsaO) and II (PsbO, PsbP, PsbW) were expressed at high levels during both the warmer period (S2) and during the colder period (S5–S7; Figure 5). During the second, colder, period the expression of hydrolases [sucrose-6-phosphate, EC 3.2.1.B3, gamma-glutamyl hydrolase, EC 3.4.19.9, S-formyl glutathione hydrolase, EC 3.1.2.12, inosine-uridine preferring nucleoside hydrolase, EC 3.2.2.1, possible alpha/beta hydrolase superfamily (lr1917 homolog), and uridine diphosphate glucose pyrophosphatase, EC 3.6.1.45], breaking larger molecules into smaller ones, were more frequent than during the initial, warmer, period (Figure 5). The expression of carbon transporters (2-oxoglutarate/malate translocator, branched-chain amino acid ABC transporter, TC 3.A.1.4.1, L-proline/glycine betaine transporter ProP) and glycerol-3-phosphate transporter, which is associated with glycolysis, occurred primarily during the colder period, and at S1 (Figure 5). The expression of hydroxymethylglutaryl-CoA lyase, EC 4.1.3.4, that is involved in the formation of ketone bodies, indicates that other carbon sources than carbohydrates were also used for metabolism, especially during the colder period (Figure 5).

### Prokaryotic Gene Expression Associated With Acquisition of Carbon

**Figure 6:**
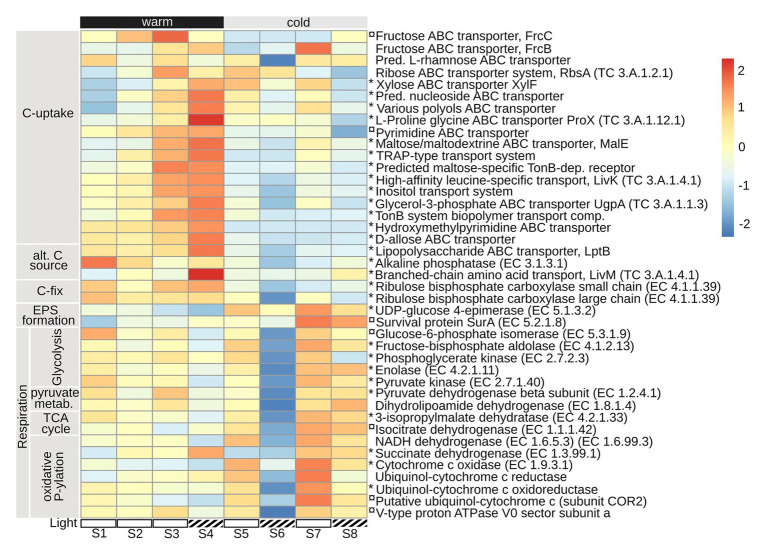
Prokaryote gene expression (excluding transcripts annotated as eukaryotes in the prokaryotic dataset) of mechanisms associated with carbon acquisition, putative extracellular polysaccharide (EPS) formation, and respiration. Included are key enzymes representative of: carbon uptake (C-uptake), alternative carbon sources, carbon fixation, EPS-formation, glycolysis, pyruvate metabolism, tricarboxylic acid (TCA) cycle, and oxidative phosphorylation. Heatmap shows the mean of triplicates per sampling date (S1–S8), normalized to TPM and square-root transformed, made using R package pheatmap, with setting scale by “row.” Warm: 19.5 ± 0.89°C, cold: 12.4 ± 1.76°C (mean temperature per 24 h in PBR); Light: light reduction, open bar → natural light, striped bar → light reduced by 40–60% by shading. * indicate *p*adj < 0.01, and ¤ *p*adj < 0.05, for log2 fold change, differential expression, contrasted for warm vs. cold time period.

Prokaryote transcription patterns suggest a lower carbon uptake during the colder period, compared to the warmer (Figure 6). During the initial, warmer, period the prokaryotes primarily expressed a range of ABC-transporters for sugars: fructose (FrcC/B), L-rhamnose, ribose (RbsA, TC 3.A.1.2.1), xylose (XylF), maltose (MalE), inositol, and allose; along with other carbohydrates, such as nucleosides, polyols, and pyrimidines (Figure 6). Transporters of alternative carbon sources were also expressed initially: lipids (lipopolysaccharide ABC transporter, LptB), and amino acids (branched-chain amino acids, LivM, TC 3.A.1.4.1, and leucine, LivK, TC 3.A.1.4.1). So was alkaline phosphatase, EC 3.1.3.1 (Figure 6), an enzyme that cleaves off inorganic phosphorus from larger organic molecules. A process which may result in the release of readily available organic carbon sources (Benitez-Nelson and Buesseler, 1999). In addition to uptake of organic carbon sources, there were indications of inorganic carbon-fixation through the expression of Rubisco small and large chain, EC 4.1.1.39, during the warmer period (Figure 6). During the colder period, especially at S7–S8, the prokaryotes expressed enzymes associated with the formation of extracellular polysaccharides (UDP-glucose 4-epimerase, EC 5.1.3.2), and for survival during stationary phase and cellular stress (survival protein SurA, EC 5.2.1.8; Degeest and De Vuyst, 2000). Enzymes involved in bacterial respiration [glycolysis, pyruvate metabolism, tricarboxylic acid-cycle (TCA), and oxidative phosphorylation] were in general expressed at higher levels during the colder period, with a clear dip in numbers at S6, compared to the warmer period (Figure 6).

### Temporal Co-Occurrences Within PBR Microbial Community

**Figure 7:**
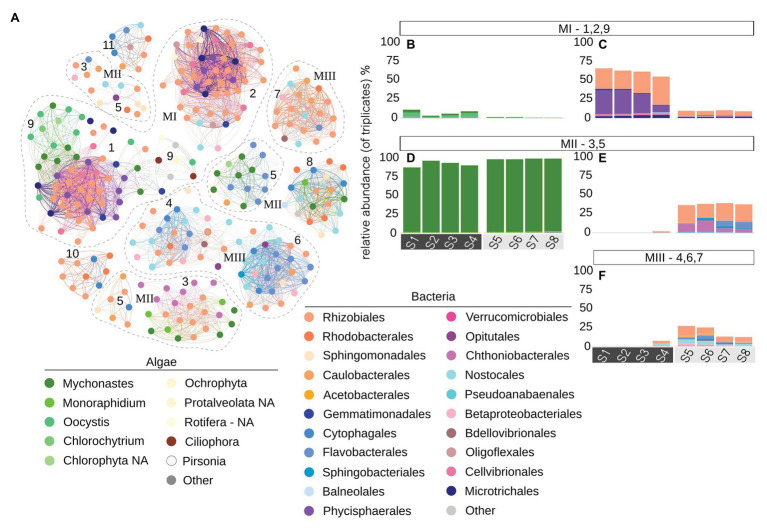
**(A)** Co-occurrence network (Pearson) of eukaryote (genus level) and prokaryote (order level) ASVs (network nodes). Only edges with weight > 0.3 were included in the plot for ease in visualization, and nodes with zero edges were excluded. Modules represent node clusters grouped based on their shared positive correlations (edges). We clustered modules into three major clusters (based on their correlation values with temperature, see Figure 8). MI (modules 1, 2, 9), MII (modules 3, 5 – spread out due to the exclusion of edges with weight < 0.3), and MIII (modules 4, 6, 7). Relative abundances of ASVs of grouped modules are displayed in **(B)** (MI – 18S), **(C)** (MI – 16S), **(D)** (MII – 18S), **(E)** (MII – 16S), and **(F)** (MIII – 16S). Bar plot facets indicate temperature regime in PBR, warm: S1–S4, cold: S5–S8.

**Figure 8:**
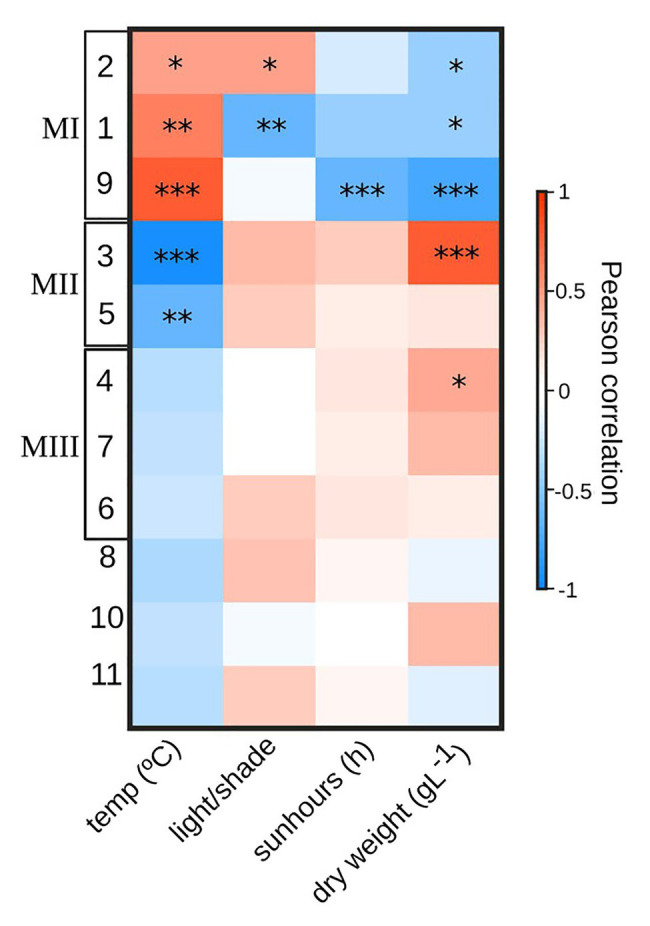
Pearson coefficient correlation values (red – positive; blue – negative) of network analysis modules with parameters: temp – temperature (°C); light/shade – induced shade (40–60% reduction of light); sunhours (h) – number of hours day^−1^ with sunshine; Dry weight – of biomass (gl^−1^). Significances indicated by *p < 0.05, **p < 0.01, and ***p ≤ 0.001. Module groups (MI, MII, and MIII) were based on shared edges (Figure 7) and similarity in correlation to temperature.

The network analysis, investigating the possible effects of temperature, light/shade, sunshine hours, and biomass on the PBR microbial community, resulted in the description of 11 modules (i.e., groups of co-occurring ASVs), of which some were interconnected with other modules by shared edges (weighted > 0.3; Figure 7A). The heatmap of Pearson correlations indicated that the temporal changes of certain ASV modules were correlated to temperature and biomass changes over time (Figure 8). Modules were grouped based on similarity in Pearson correlations together with the sharing of edges into MI – 1, 9, and 2, MII – 3 and 5, and MIII – 4, 6, and 7, for further analyses. Due to the use of cut-off values for edge weight, modules appear to spread out in the network displayed in Figure 7A, especially those in group MII. The ASVs in MI were mainly assigned to prokaryote ASVs representing the community during the initial, warmer, period (Figures 7B,C). During the colder period, the community was primarily represented by the ASVs in MII (Figures 7D,E), containing both eukaryotes and prokaryotes, and MIII, dominated by prokaryotes (Figure 7F). MI–MIII are described in more detail below.

### MI – ASVs Positively Correlated to Temperature

The interconnected modules in MI (Figure 7A) together contained 130 nodes, of which 35 were assigned to eukaryote (18S) taxa and 95 to prokaryotes (16S) taxa. Of the 18S ASVs, most were assigned to green algae of either *Mychonastes* or *Oocystis*, together representing 3–11% (relative abundance) of the microalgal community (Figure 7B) during the warmer period. The 16S ASVs were primarily assigned to Rhizobiales and Phycisphaerales, with lower levels of Gemmatimonadales, Microtrichales, Cellvibrionales, and Oligoflexales, together representing 55–65% (relative abundance) of the prokaryote community during the warmer period (Figure 7C). Signifying for all of these modules was a significant (*p* = 0.03) positive Pearson correlation (0.62 ± 0.17) with PBR temperature, and significant (*p* = 0.03) negative correlations with biomass (−0.53 ± 0.16 DW gl^−1^) and negative correlations with hours of sunshine (h; −0.40 ± 0.2), of which only those for module 9 were significant (*p* = 0.001; Figure 8).

### MII – ASVs Negatively Correlated With Temperature

The modules in MII together contained 62 nodes, of which 17 were assigned to 18S ASVs and 45 to 16S ASVs and (Figure 7A), primarily representing the community during the colder period. The 18S nodes were to large extent assigned to *Mychonastes* (including ASV_1, highly dominant during both warm and cold conditions; Figure 7D). The 16S nodes were primarily affiliated to: Rhizobiales, Bacteroidetes (Flavobacterales and Sphingobacteriales), and Chthoniobacterales, together representing 37–39% (relative abundance) of the prokaryote community during the colder period (Figure 7E) while largely absent in the warm period. Modules in MII showed significant (*p* = 0.002) negative correlation (−0.92, −0.61) with temperature in the PBR, and a significant positive correlation with biomass (0.77, *p* = 1e-05), and positive, though non-significant (*p* = 0.09) correlations with light/shade and sunshine hours (Figure 8).

### MIII – Diverse Prokaryote ASVs Associated With Cold Temperature

The modules in MIII together contained 101 nodes (Figure 7A) primarily representing the community during the colder period. This includes a single 18S node, of 0.5% relative abundance, assigned to Mychonastes while the remaining 100 nodes were assigned to a diverse set of 16S ASVs. These were annotated to orders Rhizobiales, Rhodobacterales, Sphingomonadales, Caulobacterales, Flavobacterales, Cytophagales, Nostocales, Betaproteobacteria, Opitutales, and Bdellovibrionales, together representing 12–27% (relative abundance) of the prokaryote community during the colder period (Figure 7F). MIII was significantly (*p* = 0.03) correlated to biomass, but had non-significant negative correlations (Pearson; −0.27 ± 0.04) with PBR temperature and positive correlations to light/shade and sunshine hours (Figure 8).

## Discussion

**Figure 9:**
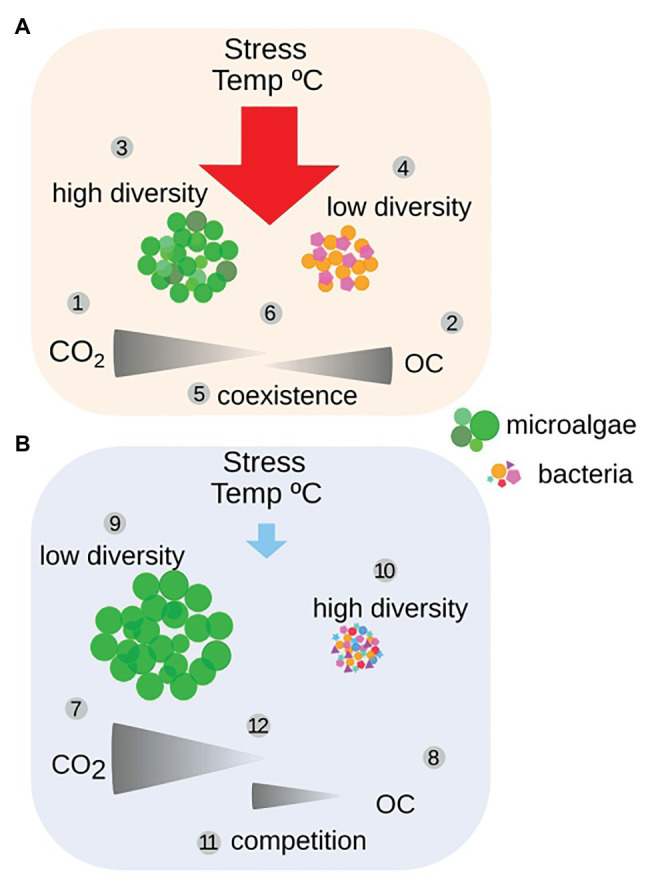
Conceptual model of the impact in microalgae-bacteria interactions induced by temperature stress **(A)**. Less CO_2_ got incorporated (1) while microalgal excretion of organic C (OC) was utilized by bacteria (2), leading to a higher diversity of the microalgae (3) and a lower diversity of the bacterial community (4). Resulting in coexistence (5), due to the partitioning of carbon resources (6). The release from temperature stress **(B)** introduced more CO_2_ to the system resulting in a higher accumulation of microalgal biomass (7), while less OC got excreted (8), leading to a lower diversity of the microalgae, being dominated by one species (9), while the bacterial diversity became higher (10). This resulted in competition between microalgae and bacteria for organic carbon (11), due to mixotrophic microalgal uptake of both CO_2_ and OC (12). The partitioning of carbon resources is indicated by CO_2_ (flue gas) and OC (autochthonously produced carbon; see Discussion for details).

It is of importance to increase our understanding of how microbial communities respond to environmental change. This can be achieved by revealing the mechanisms these communities use either to maintain their function, structure, and identity through internal adaptations or use to reform into a new type of system with new functions, structure, and identity. For aquatic microbes, this is relevant both with regard to the ecosystem services that they provide and to the impact that these changes might have on the biogeochemical cycling of nutrients in aquatic ecosystems (Daufresne and Loreau, 2001; Zell and Hubbart, 2013). The results from the present study illustrate how a PBR microbial community regain its ability to produce biomass at high capacity after having been exposed to temperature stress (during the exceptionally hot summer of 2018, 3.5°C above normal; Swedish Meteorological and Hydrological Institute), i.e., is able to respond in a resilient manner (Figure 9; Levin and Lubchenco, 2008; Feng et al., 2017). The underlying mechanisms behind this behavior are suggested to be regulated by dynamic interlevel shifts in both community structure and function, ultimately leading to interactions between eukaryotes (microalgae) and prokaryotes (bacteria) going from coexistence to competition, as seen in the regulation of uptake and utilization of organic carbon. Despite being exposed to shifts both in temperature and light, the shift in temperature was found to be the most influential structuring factor of both community structure and function (Figures 2, 8).

During the initial, warmer period, the microalgal growth was repressed, likely by heat stress, resulting in less introduced inorganic carbon through photosynthesis to the system and a significantly (*p* < 7.9e-05) lower production of biomass, and excretion of organic carbon by the microalgae (Figure 9A). Microalgal responses to abiotic stress, such as heat, include reduction in photosynthesis, as a mechanism to balance cellular energy levels necessary for metabolism (Biswal et al., 2011). Other modifications involve alterations of the cellular membrane, changes in protein and carbohydrate production, increase of cellular antioxidant and scavenge mechanisms, increased DNA-repair, as well as the induction of cell death (reviewed by Barati et al., 2019). Microalgae exposed to heat stress for a limited time have been shown to be retarded in growth, both in direct connection to the stress and up to 6h afterwards (Béchet et al., 2017). Thus, the heat stress likely induced both a lower level of photosynthesis and repressed the microalgal growth rate. The heat stress is here suspected to have opened up niches for more microalgal species, thus leading to a significantly higher diversity, *p* = 0.007, and richness, *p* = 4.4e-06, of the microalgae population (Supplementary Figure S5A). A higher diversity and richness, both in community structure and function, have been suggested to act as stabilizing factors and increase the ability of communities to be resilient to temporary disturbances (Steiner et al., 2006; Downing and Leibold, 2010; Loreau and de Mazancourt, 2013). Having a broad response diversity, a community could respond rapidly upon an environmental challenge, which could lead to the domination of one or a few species (Steiner et al., 2006). This can be exemplified by the bacterial population in the PBR where a few taxa significantly (*p* = 0.001) dominate the community during the warmer period (Figure 4 and Supplementary Figure S5B). Thus, the high availability of organic carbon appeared to have led to a lower diversity of the bacterial population structure. During the warm period, there was a partitioning of the carbon resources between microalgae and bacteria, where the microalgae primarily utilized inorganic carbon and the bacteria a range of organic carbon sources, benefitting a few groups such as Phycisphaerales and Cellvibrionales (Figure 3). Representatives from these bacterial groups have previously been found to be associated with algae. Planctomycetal Phycisphaerales was first isolated from the surface of a macroalgae (Fukunaga et al., 2009), and planctomycetal organisms have been found associated with phytoplankton biomass in the Baltic Sea (Bunse et al., 2016). Gammaproteobacterial Cellvibrionales has been shown to assimilate specific organic carbon sources, such as amino acids, glucose, and starch in coastal surface waters (Bryson et al., 2017). Indicating that a close association with an organic carbon-producing microalgae such as *Mychonastes* could be beneficial to these bacterial groups. During the colder period, the ratio of CO_2_ flow to total DW was below two, indicative of carbon limitation (Herzog and Golomb, 2004; Figure 1, Supplementary Table S7), suggesting high uptake of inorganic carbon together with a significantly higher production of microalgal biomass (*p* = 7.8e-05), which seemed to reduce the availability of organic carbon for the bacteria (Figures 6, 9B). This is indicated by a lower diversity of the microalgal population structure, favoring *Mychonastes* (Figure 7D), while there was a significant increase in bacterial diversity (*p* = 0.03; Supplementary Figure S5B). The suggested carbon limitation, leading to competition between the two levels could have been influenced by the ability of the dominant microalgal species *Mychonastes* for both C-fixation and uptake of organic carbon (Figure 5). This dual carbon utilization provides the microalgae with a competitive advantage over the bacteria, which are left to rely on respiration in order to maintain cellular processes (Figure 6). During bacterial respiration, O_2_ will be utilized and CO_2_ produced, thereby facilitating microalgal photosynthesis. This phenomenon has previously been demonstrated in laboratory co-cultures of microalgae and bacteria (Mouget et al., 1995; Danger et al., 2007). Thus, the response of the PBR microbial community upon the two different temperature conditions may have been regulated at two interconnected levels, through function (auto-, hetero-, or mixotrophy) and population structure (increased or reduced diversity), which together affect microalgae-bacteria interactions, going from coexistence to competition (Figure 9). These results suggest that the PBR microbial community, with lower complexity than natural systems, but more complex than 2-3 species model systems, has the ability to respond in a manner to temperature stress, by structural and functional modulations that span across levels, which could be considered as resilient (Supplementary Figure S1). To be resilient a community must not lose its over-all function (production of biomass), deviate from its original level of diversity, or become too different in taxonomic identity (Walker, 2004). As the interactions shift from coexistence to competition the functional guild (Vanwonterghem et al., 2014; Bryson et al., 2017) with an organic carbon preference, initially represented by bacteria become represented by both microalgae and bacteria during the colder period. This suggests that the function of organic carbon acquisition is not limited to one level, or taxonomic entity (bacteria), but may cross the inter-level boundary. Thus, the decoupling previously seen in strictly bacterial experimental systems between function and taxonomy (Fernandez et al., 2000; Wang et al., 2011; Vanwonterghem et al., 2014; Louca and Doebeli, 2016; Louca et al., 2020) is seen also in our system consisting of two levels. This underlines the importance of interlevel interactions for the ability of a community to maintain its over-all functional capacity, structure, and identity, in order to be able to respond in a resilient manner when faced with the environmental challenge (Holling, 1973).

### Theoretical Models to Describe Resilience Mechanisms

**Figure 10:**
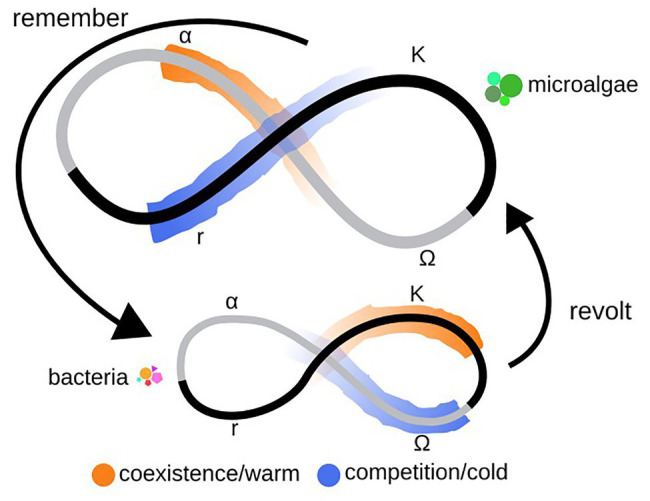
An illustration of adaptive cycles and the concept of panarchy, used to describe the interactions between the microalgae (upper level) and bacteria (lower level), going from coexistence during the warmer (orange) temperature regime to competition during the colder (blue) temperature regime. *r* – growth phase, *K* – conservation phase, *Ω* – release phase, and *α* – adaptation phase. Remember – impact on lower level by upper level, revolt – influence from lower level on upper level.

In order to study how interlevel interactions influence community resilience, the adaptive cycle model might be used (Gunderson and Holling, 2002; Walker, 2004). This cycle describes four stages that a community are thought to pass at shorter or longer intervals: birth (*r*), maturation (*K*), death (*Ω*), and renewal (*α*; Figure 10). Adaptive cycles have been used to describe the seasonal successions of algal blooms in the Baltic Sea (Angeler et al., 2015), but are more commonly applied for describing resilience in socio-ecological systems consisting of nested levels (Berkes and Ross, 2016) within the panarchy theory (Gunderson and Holling, 2002). The rationale behind the panarchy theory is that as ecosystems are made up of multiple and interconnected levels (e.g., autotrophs and heterotrophs), and each level have their own adaptive cycle, adaptations occurring at one level will influence the cycling of surrounding levels. This primarily occurs as a lower level is passing through its death/release phase, *Ω*, the “window of opportunity” during which it may collapse or start to adjust to changed conditions. If this collapse occurs when an upper level is in its least resilient phase, between birth or maturation, *r* or *K*, or in the maturation phase, it will be affected by the impact from below. When this occurs, the upper level may harbor or absorb the impact posed from below. In turn, this absorption affects the renewal/adaptation phase, *α*, of the lower level impacting the readjustments that are made to face the new conditions (Figure 10; Gunderson and Holling, 2002; Walker, 2004; Allen et al., 2014). When combining these theories with that of cross-scale resilience, that describes ecosystem resilience by functional overlaps and redundancy within and across levels (Peterson et al., 1998; Sundstrom et al., 2018), the mechanisms behind the ability of the PBR microbial community to increase its production of biomass after having been exposed to temperature stress may be explained. The cross-scale resilience model has previously been tested to describe resilience for natural ecosystems consisting of avian and mammalian populations (Wardwell et al., 2008) and of lake algae exposed to chemical waste and vertical mixing (Baho et al., 2019). Our study is the first to apply these three theories to explain the resilience of a PBR community. Here, the microalgae, representing the upper level, would – while adapting to the temperature stress during the warmer period – be somewhere in between the death/release, *Ω*, and renewal/reorganization, *α*, phases, as indicated by the more diverse microalgal population. While the bacteria – during the warmer period – would be in the steadily growing maturation/conservation, *K*, phase, as indicated by a lower population diversity (Figure 10). This suggests that the levels during this period are not posing an immediate influence on each other and are coexisting through acquiring different types of carbon (Figures 5, 6). While, during the colder period, the microalgae would have entered into r phase, becoming more structurally homogenous and starting to express new functions, and the bacteria into *Ω* phase, becoming less structurally homogenous and functionally less diverse. Leading to that the levels thus are able to have more influence on each other, according to panarchy theory. This is here ultimately represented by the evidence of competition for organic carbon manifested by microalgal expression of hydrolases for acquiring organic carbon and by a higher level of expression of bacterial transcripts associated with respiration than during the warmer period (Figures 5, 6). These expression patterns are connected to similar but opposing structural and functional adjustments among the two levels (Figure 9), where a significantly more diverse microalgal population is matched by a significantly less diverse bacterial population during the warmer period, and vice versa for the colder period (Figures 4, 9). Beyter et al. (2016) present a similar pattern in a reactor community of primarily green algae (ITS2) and bacteria (16S), where higher diversity of one coincides with lower diversity of the other during a 1year study. The combined panarchy and cross-scale resilience theories could help explain these opposing responses, saying that the response at one level help balance the response at the other level through functional overlap and redundancy (Peterson et al., 1998; Sundstrom et al., 2018). This mechanism would thus enable the maintenance of both the total structural diversity, by balancing the population diversity across the levels, and of the functional overlap in the ability for the acquisition of organic carbon found both among the bacteria and the microalgae (Figure 10; Gunderson and Holling, 2002). Thus, by applying these theoretical models, not previously used for this type of system, the regulatory mechanism by which the community responds to temperature stress may be explained (Figure 9).

### Biotic Interactions of Importance for Microbial Community Resilience

An important aspect of the concept of panarchy is the influence of interactions between levels on the resilience of a community (Gunderson and Holling, 2002). In this study, either of the two modes of interaction, coexistence or competition, dominate during a specific temperature regime linking the dynamics of interactions with the resilience of the system. When microalgae were stressed by warmer temperature, bacterial growth was promoted, leading to microalgal-bacteria coexistence, while when relieved from temperature stress the microalgal growth was promoted, and the community was governed by the competition between the levels (Figure 10). The shift in community interactions follows dynamics as proposed by Chesson (2000) in the modern coexistence theory. In which stabilizing effects of increasing niche differentiation (the use of different resources), in combination with the equalizing effects of decreasing fitness (more evenly distributed abundances) describe a situation favoring coexistence, while the opposite conditions favor competition and competitive exclusion. Examples of niche differentiation, of either light or nutrient preferences, and coexistence of different microalgal groups have been suggested by previous studies both in laboratory experiments and nature (Alexander et al., 2015; Burson et al., 2019). Studies of interactions among microbial communities often focus on niche overlaps/differentiations between similar organisms. For instance, Hunt et al. (2008) describe how members of a bacterial family in a coastal environment may coexist through resource partitioning. Previous works performed in large scale reactors with microalgae and bacteria commonly explored community stability (Stockenreiter et al., 2012; Beyter et al., 2016; Fulbright et al., 2018) rather than interlevel interactions. Interlevel interaction analysis have mostly been performed in well-designed co-cultures (Durham et al., 2014; Seyedsayamdost et al., 2014; Amin et al., 2015; Segev et al., 2016; Landa et al., 2017) or in association with natural algal blooms (Mayali et al., 2011; Teeling et al., 2012, 2016; Zhou et al., 2018), but rarely with regards to competition or coexistence (Sörenson et al., 2020). However, Le Chevanton et al. (2016) suggest that nitrogen limitation may have caused competition between algae and bacteria in a laboratory co-culture. The results from the present study suggest that interlevel interactions, in relation to functional and structural dynamics, are of importance for microbial community resilience.

### Considerations Related to our PBR Experimental Setup

This study was performed under replete nutrient conditions, enabling the focus of the study on carbon and the transfer of energy between the microalgal and bacterial populations in the PBR community. The availability of inorganic carbon was likely pushing the PBR community towards carbon limitation during the colder, more productive period, with a ratio of supplied CO_2_ to biomass at just below two (Figure 1). This is suggested by our data to have lead to the upregulation of organic carbon uptake pathways expressed by the mixotrophic microalgae (Figure 4), thus forcing the community into competition for organic carbon. In the PBR, the shift from coexistence to competition did not impact the carbon cycle flux *per se*, as the resilience of the system maintained the over-all system function, but the magnitude of cycled carbon increased as more inorganic carbon was introduced through photosynthesis during the colder period, as significantly more biomass was produced. The limited complexity in terms of community structure and influential environmental parameters of the system facilitated the analysis and allowed for the application of established ecological theories. The relatively short time scale in which the study was conducted (6weeks in total), was enough time to capture the shifts seen in response to significantly changed temperature conditions, nonetheless, more extensive sampling before and after the perturbation would have been beneficial but are not considered to limit the conclusions of this study. Models of climate change and projected environmental disturbances are based on changes seen over long periods of time (Collins and Knutti, 2013). Short-scale studies, with controlled conditions, are however important in order to reveal short-term mechanisms in microbial ecosystems, such as those seen in this study.

## Conclusions

Responses within a PBR with a mixed community of both microalgae and bacteria, when faced with changed environmental conditions, suggest that interlevel interactions, decoupling function and taxonomy, have a strong impact on the resilience of the system. The two-level system shifted from coexistence, with separate resource niches (inorganic carbon for microalgae and organic carbon for bacteria), to competition for organic carbon, with overlapping resource niches (where both microalgae and bacteria utilized organic carbon), when relieved from temperature stress. By analyzing these results with resilience theory *sensu* Holling (1973), cross-scale resilience and modern coexistence theory we may describe the mechanisms by which this system of medium complexity adapted to temperature stress through overlapping functional diversity within and functional redundancy across levels. Knowledge about these mechanisms may help improve studies related to environmental change through improved models of aquatic microbial ecosystems, and their behavior when faced with environmental perturbations.

## Data Availability Statement

The datasets presented in this study can be found in online repositories. The names of the repository/repositories and accession number(s) can be found below: https://www.ebi.ac.uk/ena, ERR3419055-ERR3419102 https://www.ebi.ac.uk/ena, ERR3421213-ERR3421260.

## Author Contributions

ES designed the study and performed sampling, laboratory, and bioinformatic work. EC contributed to methods for analysis. HF, EL, and CL contributed with interpretations and methods for analysis. ES led the writing of the manuscript together with all co-authors. All authors contributed to the article and approved the submitted version.

### Conflict of Interest

The authors declare that the research was conducted in the absence of any commercial or financial relationships that could be construed as a potential conflict of interest.
