# Supplementary material for: Temperature Stress Induces Shift From Co-Existence to Competition for Organic Carbon in Microalgae-Bacterial Photobioreactor Community – Enabling Continuous Production of Microalgal Biomass
Source: Front Microbiol. 2021 Feb 11;12:607601. doi: 10.3389/fmicb.2021.607601 (PMC7905023; doi:10.3389/fmicb.2021.607601)
Supplement: Supplementary file 1 [file Data_Sheet_1.pdf]

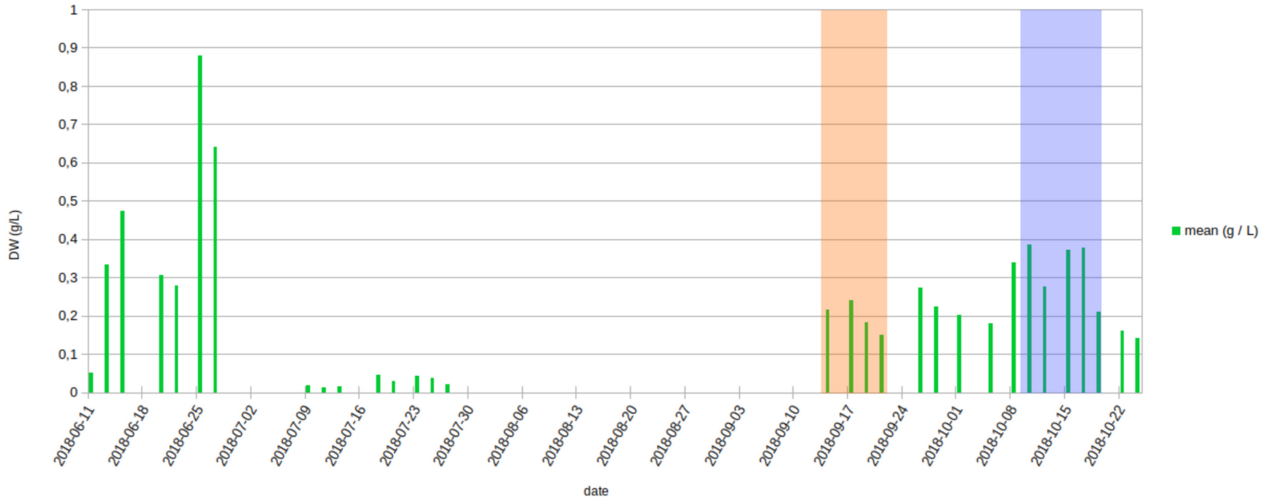

Figure S1

Production of biomass (dry weight, gL^{-1}) in PBR during June-October 2018. The two periods included in the study are shaded in orange (warmer, S1-S4) and blue (colder, S5-S8). The PBR was out of operation from Aug 3rd to Sept 10th, due to maintenance and restarted on the 11th of September with the inoculum community used during the spring, kept in laboratory conditions over the summer.

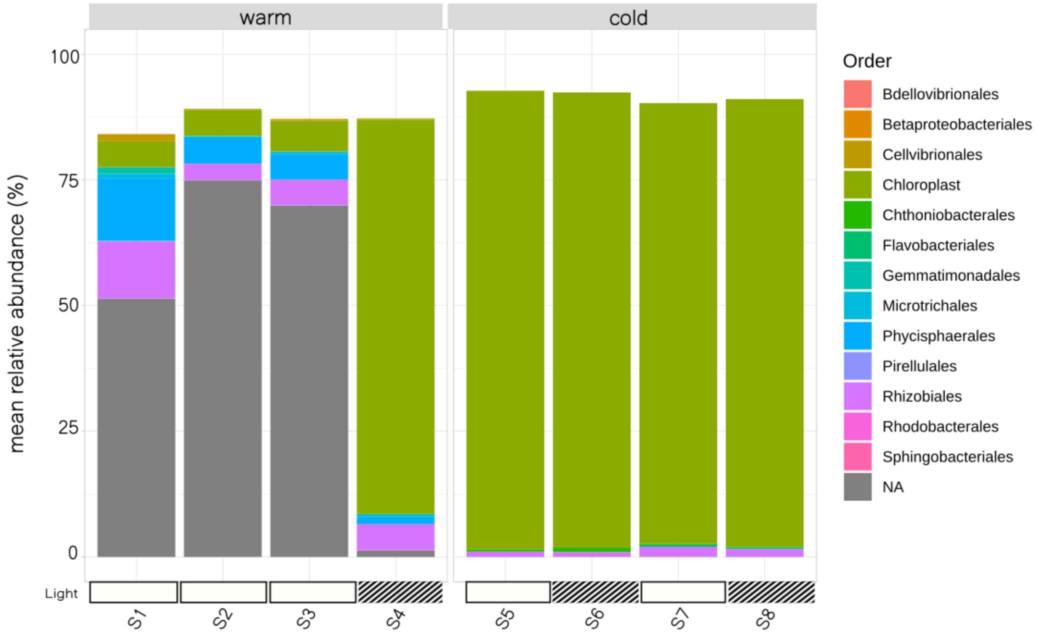

Figure S2

Relative abundance (>0.01%) of 16S ASVs, grouped at order level, including ASVs annotated as "Chloroplast". Warm: 19.5±0.89°C, cold: 12.4±1.76°C; Light: light reduction, open bar → natural light, striped bar → light reduced by 40-60% by shading.

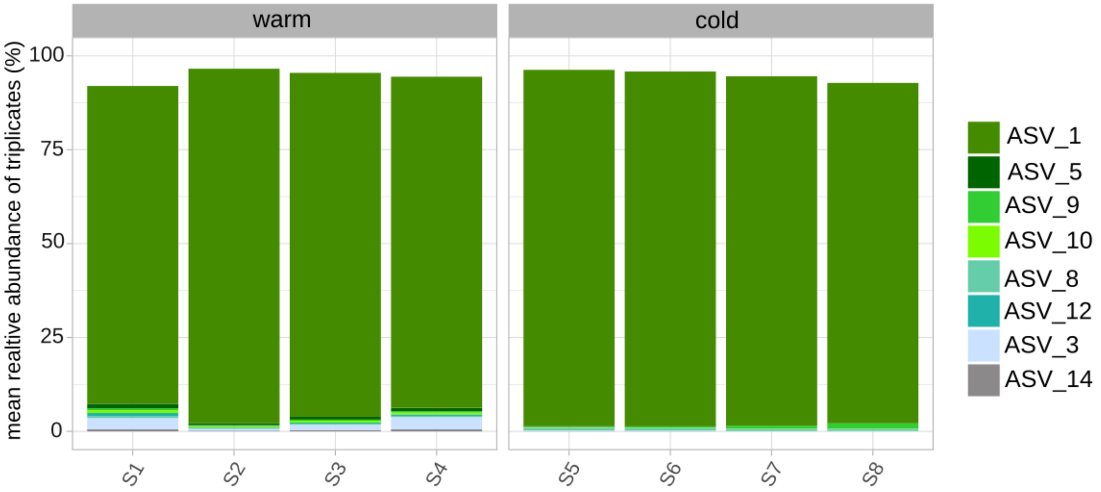

Figure S3

Relative abundances (%) of top 8 18S ASVs.

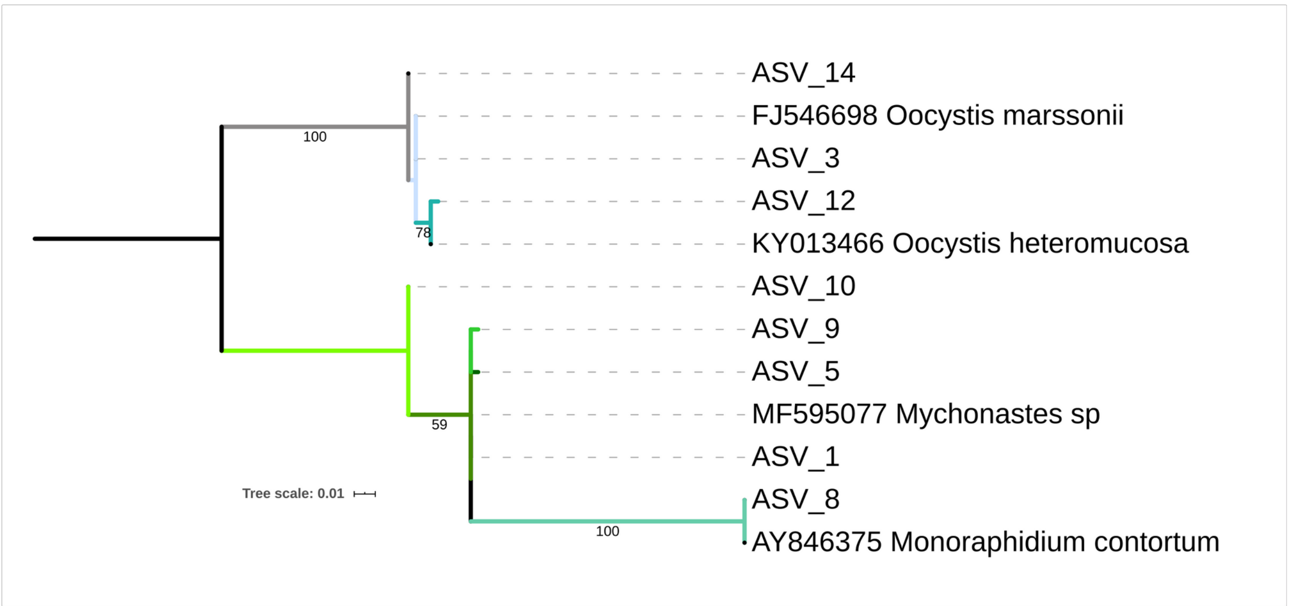

Figure S4

Maximum likelihood tree (500 bootstraps) of top 8 18S ASVs with references (given with GenBank accession numbers).

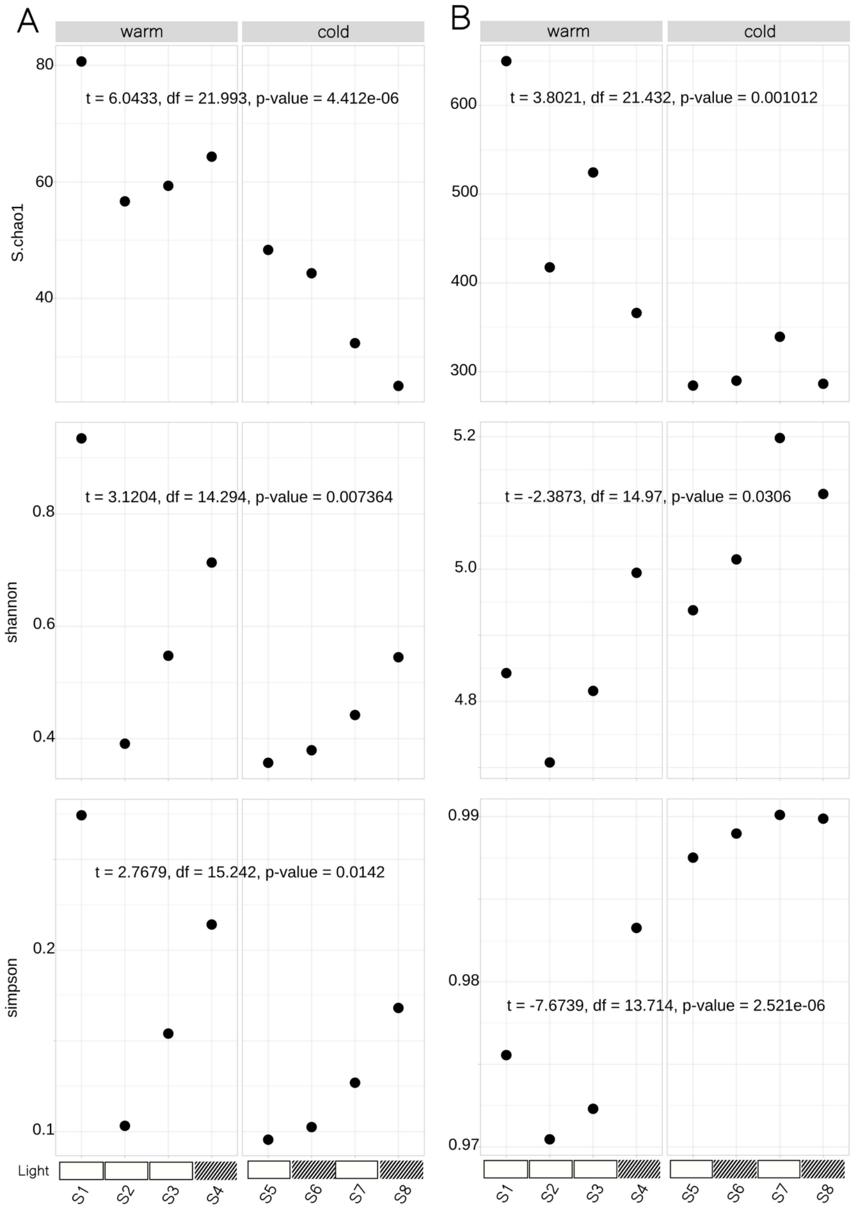

Figure S5

Diversity indices for rarefied ASVs data. A) 18S and B) 16S. Top panels - species richness (S.Chao1 Index). Diversity and evenness indices Shannon – middles panel, Simpson - lower panels. Values represent mean of triplicates per sampled date (S1-S8). Results from t-tests contrasting warm and cold period are shown for each index. Warm: 19.5±0.89°C, cold: 12.4±1.76°C (mean temperature per 24h in PBR). Light: light reduction, open bar → natural light, striped bar → light reduced by 40-60% by shading.

Figure S6

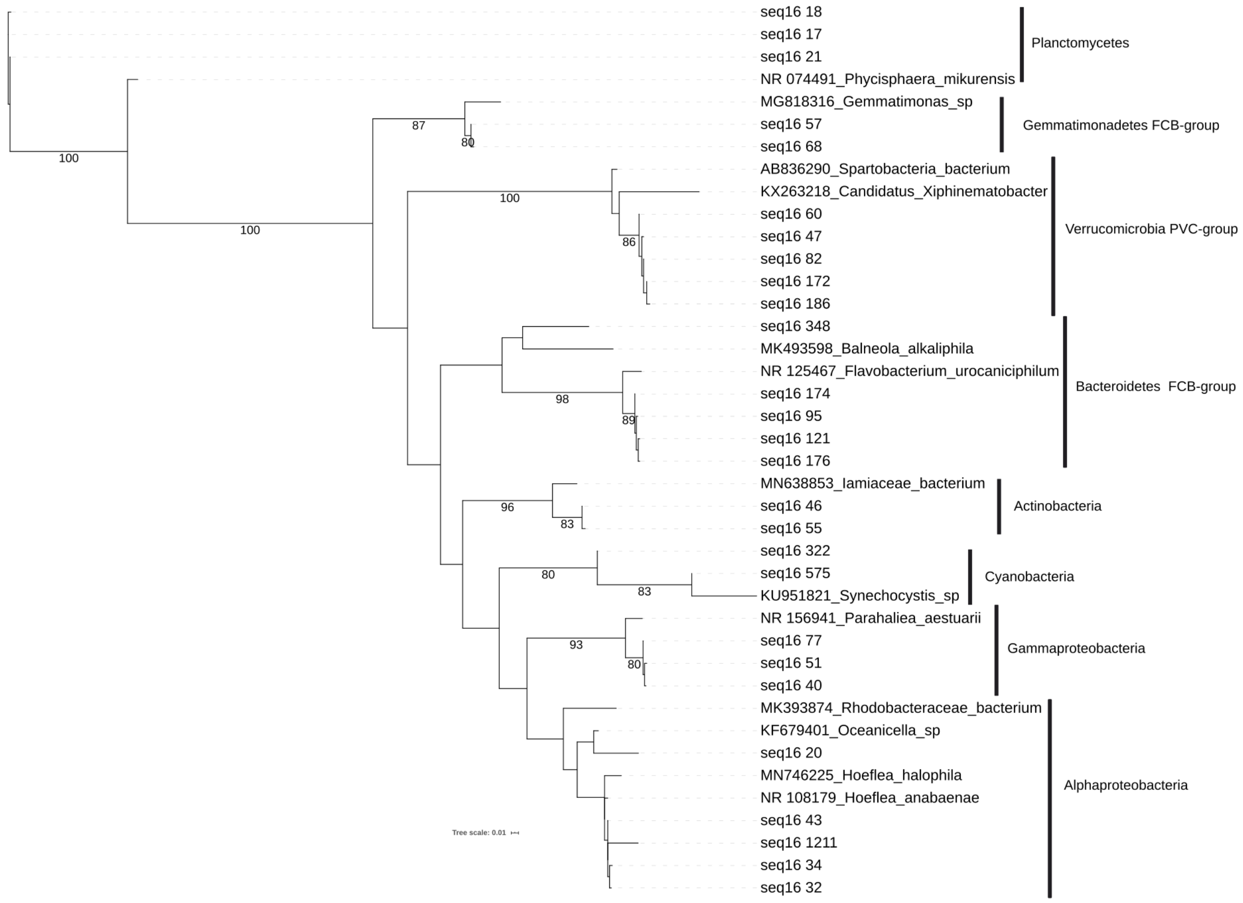

Maximum likelihood tree of representative 16S ASV (seq16) with references (given with GenBank accession numbers). Bootstraps (x100) given as branch support (>75).

Sample	Day	Date	Reads in	Reads out
**25**	**Sept 12**	**S1**	374265	274448
**26**	**Sept 12**	**S1**	350549	257887
**27**	**Sept 12**	**S1**	285873	213991
**28**	**Sept 14**	**S2**	401778	303885
**29**	**Sept 14**	**S2**	338785	254066
**30**	**Sept 14**	**S2**	326424	250774
**31**	**Sept 19**	**S3**	233946	176965
**32**	**Sept 19**	**S3**	367827	274139
**33**	**Sept 19**	**S3**	347488	275843
**34**	**Sept 21**	**S4**	267967	209930
**35**	**Sept 21**	**S4**	313518	250876
**36**	**Sept 21**	**S4**	311506	245627
**37**	**Oct 10**	**S5**	344532	269539
**38**	**Oct 10**	**S5**	318521	253096
**39**	**Oct 10**	**S5**	286531	227141
**40**	**Oct 12**	**S6**	182725	145611
**41**	**Oct 12**	**S6**	325340	255374
**42**	**Oct 12**	**S6**	208083	161558
**43**	**Oct 17**	**S7**	160664	124462
**44**	**Oct 17**	**S7**	161825	123597
**45**	**Oct 17**	**S7**	174313	131856
**46**	**Oct 19**	**S8**	186968	137797
**47**	**Oct 19**	**S8**	70166	52363
**48**	**Oct 19**	**S8**	94935	71504
		**sum**	6,464,529	4,942,329

Table S1

Number of 18S reads before and after error model filtration in Dada2.

Sample	Day	Date	Reads in	Reads out
**1**	**Sept 12**	**S1**	808187	689815
**2**	**Sept 12**	**S1**	327766	278734
**3**	**Sept 12**	**S1**	768249	661398
**4**	**Sept 14**	**S2**	676875	595100
**5**	**Sept 14**	**S2**	549166	483339
**6**	**Sept 14**	**S2**	587707	516290
**7**	**Sept 19**	**S3**	594776	520788
**8**	**Sept 19**	**S3**	773976	679125
**9**	**Sept 19**	**S3**	709125	623360
**10**	**Sept 21**	**S4**	413082	346514
**11**	**Sept 21**	**S4**	427326	364445
**12**	**Sept 21**	**S4**	482065	404759
**13**	**Oct 10**	**S5**	551337	485704
**14**	**Oct 10**	**S5**	766976	667436
**15**	**Oct 10**	**S5**	410276	363261
**16**	**Oct 12**	**S6**	429100	380364
**17**	**Oct 12**	**S6**	471722	410809
**18**	**Oct 12**	**S6**	604047	526087
**19**	**Oct 17**	**S7**	342827	299926
**20**	**Oct 17**	**S7**	554677	476978
**21**	**Oct 17**	**S7**	451738	391329
**22**	**Oct 19**	**S8**	486301	422618
**23**	**Oct 19**	**S8**	383886	336289
**24**	**Oct 19**	**S8**	410731	358723
		**sum**	12,981,918	11,283,191

Table S2

Number of 16S reads before and after error model filtration in Dada2.

Sum total reads before and after filtration.

UK						
**Sample**	**FastQ input**	**FastQ pairs kept**	**FastQ pairs discarded**	**% kept**	**FastQ singles kept**	**FastQ singles discarded**
**ALB_0912_A**	27095782	25837582	76950	95.4	590625	590625
**ALB_0912_B**	38350200	35350646	28814	92.2	1485370	1485370
**ALB_0912_C**	26850774	24548746	29350	91.4	1136339	1136339
**ALB_0914_A**	35339164	33670754	14720	95.3	826845	826845
**ALB_0914_B**	51876714	48781534	24238	94.0	1535471	1535471
**ALB_0914_C**	49898626	47502564	41510	95.2	1177276	1177276
**ALB_0919_A**	46116074	41840170	18700	90.7	2128602	2128602
**ALB_0919_B**	49321144	46309092	15276	93.9	1498388	1498388
**ALB_0919_C**	37188998	34479848	24472	92.7	1342339	1342339
**ALB_0921_A**	43247320	40368796	20032	93.3	1429246	1429246
**ALB_0921_B**	46354204	44210302	15864	95.4	1064019	1064019
**ALB_0921_C**	33147572	30887272	21340	93.2	1119480	1119480
**ALB_1010_A**	38977170	36498840	15824	93.6	1231253	1231253
**ALB_1010_B**	45872386	43720788	23788	95.3	1063905	1063905
**ALB_1010_C**	46033112	43266312	23382	94.0	1371709	1371709
**ALB_1012_A**	44236476	40723784	24734	92.1	1743979	1743979
**ALB_1012_B**	42604218	39661296	28380	93.1	1457271	1457271
**ALB_1012_C**	52239078	49779232	17774	95.3	1221036	1221036
**ALB_1017_A**	47089326	44078178	23454	93.6	1493847	1493847
**ALB_1017_B**	45841126	42839844	45916	93.5	1477683	1477683
**ALB_1017_C**	45181670	41453032	49188	91.7	1839725	1839725
**ALB_1019_A**	45104008	42156930	63708	93.5	1441685	1441685
**ALB_1019_B**	38104268	35617798	20124	93.5	1233173	1233173
**ALB_1019_C**	42999454	39994068	17488	93.0	1493949	1493949

Table S3

Processing of raw eukaryote (Poly-A selected) metatranscriptome reads using FastQC.

PRO						
**Sample**	**FastQ input**	**FastQ pairs kept**	**FastQ pairs discarded**	**% kept**	**FastQ singles kept**	**FastQ singles discarded**
**ALB_0912_A**	41181864	40806370	3742	99.1	185876	185876
**ALB_0912_B**	41003308	40647116	3016	99.1	176588	176588
**ALB_0912_C**	38051228	37783548	4578	99.3	131551	131551
**ALB_0914_A**	47029166	46708808	2596	99.3	158881	158881
**ALB_0914_B**	43169622	42523444	4196	98.5	320991	320991
**ALB_0914_C**	40588450	40313242	2238	99.3	136485	136485
**ALB_0919_A**	42124356	41828366	4036	99.3	145977	145977
**ALB_0919_B**	40839750	40410594	7008	98.9	211074	211074
**ALB_0919_C**	42042600	41796694	6402	99.4	119752	119752
**ALB_0921_A**	42190932	41812134	3224	99.1	187787	187787
**ALB_0921_B**	36978322	36389630	2894	98.4	292899	292899
**ALB_0921_C**	36542306	36071504	1704	98.7	234549	234549
**ALB_1010_A**	42771842	42516536	2118	99.4	126594	126594
**ALB_1010_B**	42629362	42457040	2992	99.6	84665	84665
**ALB_1010_C**	35978182	35812782	3280	99.5	81060	81060
**ALB_1012_A**	37620952	37030526	1528	98.4	294449	294449
**ALB_1012_B**	37159272	36811514	1526	99.1	173116	173116
**ALB_1012_C**	36557422	36340872	2312	99.4	107119	107119
**ALB_1017_A**	45961236	45687742	3274	99.4	135110	135110
**ALB_1017_B**	37651982	37479582	3266	99.5	84567	84567
**ALB_1017_C**	44319618	44158808	6564	99.6	77123	77123
**ALB_1019_A**	43181032	42902548	5174	99.4	136655	136655
**ALB_1019_B**	37714036	37484900	3282	99.4	112927	112927
**ALB_1019_C**	33152668	33039020	2188	99.7	55730	55730

Table S4

Processing of raw non Poly-A selected metatranscriptome reads using FastQC.

Sample	Dry weight (gL^{-1})	Std	Dry weight total (3200 L)	Mass CO_2 added (g)	Mass CO_2:DW_{tot}
**S1**	na	na	na	na	na
**S2**	0.22	±0.005	704	3126	4.4
**S3**	0.18	±0.049	576	1992	3.5
**S4**	0.15	±0.02	480	2340	4.9
**S5**	0.39	±0.06	1248	2255	1.8
**S6**	0.28	±0.03	896	2088	2.3
**S7**	0.38	±0.08	1216	2350	1.9
**S8**	0.21	±0.03	672	1219	1.8

Table S7

Measured algal biomass in dry weight (gL^{-1}). Total dry weight in reactor (total 3200 L). Mass of CO_2 flow into the system (g). The ratio of CO_2 added to the system and total dry weight (Dw_{tot}).
