# Supplementary material for: Temperature Stress Induces Shift From Co-Existence to Competition for Organic Carbon in Microalgae-Bacterial Photobioreactor Community – Enabling Continuous Production of Microalgal Biomass
Source: Front Microbiol. 2021 Feb 11;12:607601. doi: 10.3389/fmicb.2021.607601 (PMC7905023; doi:10.3389/fmicb.2021.607601)
Supplement: Supplementary file 2 [file Data_Sheet_2.pdf]

| Date | $NH_4$ (µM) | std | NOx ($NO_3$+$NO_2$) (µM) | std | Inorganic N (µM) | | TN (µM) |
|---|---|---|---|---|---|---|---|
| **S1** | na | na | na | na | na | na | na |
| **S2** | 1.13 | ±0.002 | 1045 | ±0.02 | 1046 | | 1327 |
| **S3** | 1.44 | ±0.001 | 1090 | ±0.05 | 1091 | | 1308 |
| **S4** | 2.05 | ±0.012 | 1579 | ±0.005 | 1579 | | 1803 |
| **S5** | n.a. | n.a. | 1139 | ±0.01 | n.a. | | 1947 |
| **S6** | 1.26 | ±0.004 | 1011 | ±0.05 | 1013 | | 1169 |
| **S7** | 0.95 | ±0.002 | 401 | ±0.05 | 402 | | 585 |
| **S8** | 1.03 | ±0.001 | 835 | ±0.07 | 836 | | 880 |
| **Date** | **$PO_4$ (µM)** | **std** | **TP (µM)** | **std** | **cDOM** | **std** | **TN:TP** |
| **S1** | na | na | na | na | na | na | na |
| **S2** | 0.10 | ±0.0007 | 0.53 | ±0 | 1.70 | ±0.011 | 2527:1 |
| **S3** | 1.38 | ±0.0042 | 1.83 | ±0.02 | 2.29 | ±0.016 | 714:1 |
| **S4** | 0.10 | ±0 | 0.66 | ±0.03 | 2.86 | ±0.006 | 2726:1 |
| **S5** | 0.10 | ±0.0021 | 0.50 | ±0.01 | 1.74 | ±0.029 | 3868:1 |
| **S6** | 0.12 | ±0.0007 | 0.47 | ±0.02 | 1.22 | ±0.017 | 2500:1 |
| **S7** | 0.15 | ±0.0028 | 0.39 | ±0.03 | 1.03 | ±0.011 | 1505:1 |
| **S8** | 0.11 | ±0.0007 | 0.40 | ±0.04 | 1.60 | ±0.009 | 2224:1 |

*Supporting Information T1*

*Concentrations of ammonium ($NH_4$ µM), nitrate and nitrite ($NO_3$+$NO_2$ µM), Inorganic Nitrogen (µM), Total N (TN µM), Phosphate ($PO_4$ µM), Total P (µM), Colored Dissolved Organic Matter (cDOM 440 nm absorbance), in the photobioreactor (PBR) water from each sampling date. S1 denoted by* na *as no measurements were made on that date. Measurements represent mean of duplicates or triplicates.*
