# Supplementary material for: Temperature Stress Induces Shift From Co-Existence to Competition for Organic Carbon in Microalgae-Bacterial Photobioreactor Community – Enabling Continuous Production of Microalgal Biomass
Source: Front Microbiol. 2021 Feb 11;12:607601. doi: 10.3389/fmicb.2021.607601 (PMC7905023; doi:10.3389/fmicb.2021.607601)
Supplement: Supplementary file 3 [file Data_Sheet_3.pdf]

Supplementary Table S5

ORF	Rank3	Log2Fold Change	base Mean	lfcSE	padj
k141_548983	2-oxoglutarate/malate translocator	-1,53	1387,78	0,15	2,6174E-21
k141_333091	2-oxoglutarate/malate translocator	-1,69	101,41	0,18	3,84763E-20
k141_475239	2-oxoglutarate/malate translocator	-1,45	136,58	0,19	1,52129E-13
k141_102628	2-oxoglutarate/malate translocator	2,13	90,94	0,28	9,88886E-13
k141_117083	2-oxoglutarate/malate translocator	-0,93	18863,85	0,15	1,31208E-08
k141_452363	2-oxoglutarate/malate translocator	-1,48	32,04	0,25	5,06933E-08
k141_179996	2-oxoglutarate/malate translocator	2,33	71,88	0,41	1,57078E-07
k141_371041	2-oxoglutarate/malate translocator	-0,86	4174,34	0,15	2,25676E-07
k141_327423	2-oxoglutarate/malate translocator	2,22	18,80	0,46	8,48587E-06
k141_200378	2-oxoglutarate/malate translocator	-1,60	21,40	0,35	3,12594E-05
k141_262791	2-oxoglutarate/malate translocator	1,41	356,90	0,31	3,23282E-05
k141_241719	2-oxoglutarate/malate translocator	1,79	14,58	0,43	0,000128015
k141_260463	2-oxoglutarate/malate translocator	3,47	2,84	0,87	0,000273131
k141_266022	2-oxoglutarate/malate translocator	-0,88	41,89	0,22	0,00028495
k141_408987	2-oxoglutarate/malate translocator	2,24	4,02	0,59	0,000532837
k141_102291	2-oxoglutarate/malate translocator	-2,68	4,39	0,72	0,000768286
k141_319289	Branched-chain amino acid ABC transporter, amino acid-binding protein (TC 3.A.1.4.1)	-1,69	31,18	0,22	6,90317E-13
k141_156650	Carbonic anhydrase (EC 4.2.1.1)	2,74	53,82	0,24	3,91823E-29
k141_354975	Carbonic anhydrase (EC 4.2.1.1)	2,62	170,96	0,26	1,49879E-22
k141_214223	Carbonic anhydrase (EC 4.2.1.1)	6,84	10,28	0,73	2,12509E-19
k141_211486	Carbonic anhydrase (EC 4.2.1.1)	-6,55	16,54	0,76	2,47027E-16
k141_143280	Carbonic anhydrase (EC 4.2.1.1)	1,79	174,39	0,22	1,62045E-14
k141_495726	Carbonic anhydrase (EC 4.2.1.1)	5,14	36,48	0,64	2,63411E-14
k141_356359	Carbonic anhydrase (EC 4.2.1.1)	-6,00	11,44	0,75	3,68066E-14
k141_303879	Carbonic anhydrase (EC 4.2.1.1)	-4,03	16,80	0,56	7,20381E-12
k141_414077	Carbonic anhydrase (EC 4.2.1.1)	5,29	5,02	0,78	1,28543E-10
k141_459441	Carbonic anhydrase (EC 4.2.1.1)	1,46	42,79	0,24	1,26296E-08
k141_216585	Carbonic anhydrase (EC 4.2.1.1)	4,66	16,41	0,82	1,19559E-07
k141_363238	Carbonic anhydrase (EC 4.2.1.1)	4,95	2,77	0,87	1,20493E-07
k141_531878	Carbonic anhydrase (EC 4.2.1.1)	1,70	36,90	0,30	1,2272E-07
k141_542525	Carbonic anhydrase (EC 4.2.1.1)	4,26	4,67	0,79	4,75144E-07
k141_149506	Carbonic anhydrase (EC 4.2.1.1)	5,28	3,47	0,98	5,25191E-07
k141_303356	Carbonic anhydrase (EC 4.2.1.1)	-1,93	14,24	0,37	1,09638E-06
k141_286844	Carbonic anhydrase (EC 4.2.1.1)	4,55	4,22	0,89	1,93903E-06
k141_434110	Carbonic anhydrase (EC 4.2.1.1)	-3,47	47,22	0,68	2,35356E-06
k141_184675	Carbonic anhydrase (EC 4.2.1.1)	3,89	15,47	0,78	3,86526E-06
k141_444418	Carbonic anhydrase (EC 4.2.1.1)	3,35	16,91	0,68	6,09955E-06
k141_157322	Carbonic anhydrase (EC 4.2.1.1)	3,66	5,77	0,76	9,13241E-06
k141_328623	Carbonic anhydrase (EC 4.2.1.1)	1,40	187,89	0,29	1,12499E-05
k141_367283	Carbonic anhydrase (EC 4.2.1.1)	4,08	7,31	0,86	1,20994E-05
k141_459203	Carbonic anhydrase (EC 4.2.1.1)	4,05	5,53	0,86	1,26198E-05
k141_314915	Carbonic anhydrase (EC 4.2.1.1)	2,16	9,28	0,46	1,573E-05
k141_289263	Carbonic anhydrase (EC 4.2.1.1)	-2,85	4,38	0,61	1,90999E-05
k141_96334	Carbonic anhydrase (EC 4.2.1.1)	4,77	2,45	1,03	2,09178E-05
k141_68948	Carbonic anhydrase (EC 4.2.1.1)	1,82	687,89	0,40	2,70967E-05
k141_484827	Carbonic anhydrase (EC 4.2.1.1)	4,55	3,01	1,00	2,96163E-05
k141_464700	Carbonic anhydrase (EC 4.2.1.1)	3,16	7,23	0,70	3,10582E-05
k141_9518	Carbonic anhydrase (EC 4.2.1.1)	4,72	2,35	1,07	4,99506E-05
k141_318302	Carbonic anhydrase (EC 4.2.1.1)	-3,55	5,64	0,81	5,17727E-05

Supplementary Table S5

ORF	Rank3	Log2Fold Change	base Mean	lfcSE	padj
**k141_3059**	**Carbonic anhydrase (EC 4.2.1.1)**	4,62	2,21	1,07	7,43748E-05
**k141_150193**	**Carbonic anhydrase (EC 4.2.1.1)**	4,65	2,25	1,09	8,83906E-05
**k141_369195**	**Carbonic anhydrase (EC 4.2.1.1)**	3,92	9,00	0,95	0,000169426
**k141_566540**	**Carbonic anhydrase (EC 4.2.1.1)**	3,45	5,94	0,84	0,000171725
**k141_94851**	**Carbonic anhydrase (EC 4.2.1.1)**	1,57	16,71	0,39	0,000203793
**k141_213823**	**Carbonic anhydrase (EC 4.2.1.1)**	3,82	4,48	0,96	0,000282481
**k141_500524**	**Carbonic anhydrase (EC 4.2.1.1)**	4,79	3,58	1,21	0,000318637
**k141_178359**	**Carbonic anhydrase (EC 4.2.1.1)**	4,80	2,47	1,22	0,000338775
**k141_40985**	**Carbonic anhydrase (EC 4.2.1.1)**	-4,62	2,22	1,19	0,000389359
**k141_45252**	**Carbonic anhydrase (EC 4.2.1.1)**	2,44	6,52	0,63	0,000418807
**k141_198190**	**Carbonic anhydrase (EC 4.2.1.1)**	-2,46	8,36	0,64	0,00050449
**k141_328628**	**Carbonic anhydrase (EC 4.2.1.1)**	4,73	2,35	1,24	0,000549087
**k141_290108**	**Carbonic anhydrase (EC 4.2.1.1)**	3,50	5,81	0,92	0,000558005
**k141_21358**	**Carbonic anhydrase (EC 4.2.1.1)**	3,52	4,85	0,94	0,000681963
**k141_117901**	**Carbonic anhydrase (EC 4.2.1.1)**	3,67	2,35	1,00	0,000828477
**k141_547573**	**Carbonic anhydrase (EC 4.2.1.1)**	4,94	2,72	1,34	0,000862505
**k141_147667**	**Carbonic anhydrase (EC 4.2.1.1)**	4,40	1,88	1,23	0,001199178
**k141_273287**	**Carbonic anhydrase (EC 4.2.1.1)**	-4,38	1,88	1,25	0,001479676
**k141_126164**	**Carbonic anhydrase (EC 4.2.1.1)**	4,27	1,72	1,23	0,001669531
**k141_219560**	**Carbonic anhydrase (EC 4.2.1.1)**	-5,04	2,97	1,49	0,002319709
**k141_297216**	**Carbonic anhydrase (EC 4.2.1.1)**	4,12	2,22	1,24	0,002657057
**k141_392312**	**Carbonic anhydrase (EC 4.2.1.1)**	3,51	1,50	1,11	0,004632651
**k141_297940**	**Carbonic anhydrase (EC 4.2.1.1)**	3,88	1,31	1,24	0,004884004
**k141_365333**	**Carbonic anhydrase (EC 4.2.1.1)**	-4,22	1,67	1,37	0,005709171
**k141_568317**	**Carbonic anhydrase (EC 4.2.1.1)**	4,14	1,57	1,37	0,006635881
**k141_176042**	**Carbonic anhydrase (EC 4.2.1.1)**	-1,75	5,42	0,58	0,007161562
**k141_273684**	**Carbonic anhydrase (EC 4.2.1.1)**	4,04	1,47	1,40	0,009697377
**k141_212502**	**Gamma-glutamyl hydrolase (EC 3.4.19.9)**	6,10	6,11	0,77	6,78516E-14
**k141_353437**	**Gamma-glutamyl hydrolase (EC 3.4.19.9)**	-1,90	45,80	0,34	2,20347E-07
**k141_127527**	**Gamma-glutamyl hydrolase (EC 3.4.19.9)**	-1,45	18,35	0,39	0,000599484
**k141_74774**	**Gamma-glutamyl hydrolase (EC 3.4.19.9)**	4,29	1,74	1,38	0,005247935
**k141_481081**	**Glycerol-3-phosphate transporter**	2,23	265,17	0,24	3,95873E-19
**k141_224399**	**Glycerol-3-phosphate transporter**	2,01	40,10	0,25	4,92828E-14
**k141_193668**	**Glycerol-3-phosphate transporter**	2,32	18,62	0,33	2,22057E-11
**k141_389014**	**Glycerol-3-phosphate transporter**	-0,31	3154,59	0,08	0,000447475
**k141_457268**	**Glycerol-3-phosphate transporter**	4,49	2,02	1,23	0,000895182
**k141_425508**	**Glycerol-3-phosphate transporter**	-1,53	10,83	0,42	0,000997363
**k141_146839**	**Hydroxymethylglutaryl-CoA lyase (EC 4.1.3.4)**	4,68	6,52	0,90	1,5418E-06
**k141_139861**	**Hydroxymethylglutaryl-CoA lyase (EC 4.1.3.4)**	-1,13	2051,92	0,22	2,22582E-06
**k141_502140**	**Hydroxymethylglutaryl-CoA lyase (EC 4.1.3.4)**	-4,41	1,93	1,14	0,000425189
**k141_510582**	**Hydroxymethylglutaryl-CoA lyase (EC 4.1.3.4)**	1,39	15,18	0,42	0,002557706
**k141_230741**	**Hydroxymethylglutaryl-CoA lyase (EC 4.1.3.4)**	2,11	2,96	0,72	0,009191594
**k141_125817**	**Inosine-uridine preferring nucleoside**	-0,60	8875,48	0,09	1,2322E-10
**k141_34024**	**Inosine-uridine preferring nucleoside**	4,51	7,55	0,73	6,58206E-09
**k141_67379**	**Inosine-uridine preferring nucleoside**	-5,27	3,48	1,07	5,37464E-06
**k141_237094**	**Inosine-uridine preferring nucleoside**	2,52	9,44	0,55	2,58737E-05
**k141_357842**	**Inosine-uridine preferring nucleoside**	-3,92	2,78	0,89	5,04037E-05
**k141_543936**	**Inosine-uridine preferring nucleoside**	4,29	2,46	0,97	5,42924E-05
**k141_1288**	**Inosine-uridine preferring nucleoside**	4,01	2,92	1,00	0,000277133
**k141_253294**	**Inosine-uridine preferring nucleoside**	3,33	9,72	1,04	0,003892242

Supplementary Table S5

ORF	Rank3	Log2Fold Change	base Mean	lfcSE	padj
k141_76955	Inosine-uridine preferring nucleoside	2,81	5,23	0,97	0,009333198
k141_355623	L-Proline/Glycine betaine transporter ProP	3,92	9,01	0,79	3,90087E-06
k141_65112	L-Proline/Glycine betaine transporter ProP	3,28	12,83	0,83	0,000325879
k141_215212	L-Proline/Glycine betaine transporter ProP	3,79	4,76	1,08	0,001444126
k141_559550	L-Proline/Glycine betaine transporter ProP	-4,11	1,55	1,22	0,002426106
k141_498191	L-Proline/Glycine betaine transporter ProP	-1,99	3,46	0,68	0,00891984
k141_416324	photosystem I subunit II (PsaD)	2,58	413,91	0,21	3,74569E-32
k141_535769	photosystem I subunit II (PsaD)	-1,54	443,63	0,13	4,10825E-29
k141_199899	photosystem I subunit II (PsaD)	7,44	74,50	0,66	1,26862E-27
k141_247909	photosystem I subunit II (PsaD)	-2,51	72,70	0,29	7,72722E-17
k141_565346	photosystem I subunit II (PsaD)	-1,33	170,46	0,17	5,50027E-14
k141_235587	photosystem I subunit II (PsaD)	1,98	1351,95	0,27	3,54144E-12
k141_73751	photosystem I subunit II (PsaD)	1,60	28,00	0,27	2,60764E-08
k141_266241	photosystem I subunit II (PsaD)	-1,44	58,85	0,24	3,80212E-08
k141_566820	photosystem I subunit II (PsaD)	2,94	7,21	0,58	2,99159E-06
k141_445585	photosystem I subunit II (PsaD)	-0,57	160,33	0,16	0,001142777
k141_104668	photosystem I subunit II (PsaD)	3,20	2,45	0,94	0,002100047
k141_207282	photosystem I subunit II (PsaD)	3,41	3,43	1,12	0,006501858
k141_98502	photosystem I subunit III precursor, plastocyanin (cyt c553) docking protein (PsaF)	2,38	403,48	0,22	6,02003E-25
k141_93528	photosystem I subunit III precursor, plastocyanin (cyt c553) docking protein (PsaF)	2,00	761,02	0,27	1,77381E-12
k141_208417	photosystem I subunit III precursor, plastocyanin (cyt c553) docking protein (PsaF)	-0,72	742,75	0,10	6,38572E-11
k141_464550	photosystem I subunit III precursor, plastocyanin (cyt c553) docking protein (PsaF)	-1,54	47,06	0,23	1,44424E-10
k141_522821	photosystem I subunit III precursor, plastocyanin (cyt c553) docking protein (PsaF)	-2,65	36,44	0,41	1,28445E-09
k141_295117	photosystem I subunit III precursor, plastocyanin (cyt c553) docking protein (PsaF)	1,75	845,12	0,29	2,57856E-08
k141_398369	photosystem I subunit III precursor, plastocyanin (cyt c553) docking protein (PsaF)	-1,40	103,70	0,24	7,4738E-08
k141_3413	photosystem I subunit III precursor, plastocyanin (cyt c553) docking protein (PsaF)	-0,66	295,07	0,12	9,42142E-08
k141_332077	photosystem I subunit III precursor, plastocyanin (cyt c553) docking protein (PsaF)	-1,56	239,84	0,31	4,74773E-06
k141_138596	photosystem I subunit III precursor, plastocyanin (cyt c553) docking protein (PsaF)	2,53	5,46	0,60	0,000121784
k141_577144	photosystem I subunit III precursor, plastocyanin (cyt c553) docking protein (PsaF)	-1,77	8,68	0,49	0,000912868
k141_138301	photosystem I subunit III precursor, plastocyanin (cyt c553) docking protein (PsaF)	3,11	2,34	0,86	0,001030409
k141_87224	photosystem I subunit III precursor, plastocyanin (cyt c553) docking protein (PsaF)	2,44	3,24	0,78	0,005159722
k141_13050	photosystem I subunit PsaO	2,58	199,31	0,21	4,83136E-32
k141_432749	photosystem I subunit PsaO	-1,12	441,89	0,12	2,82635E-20
k141_578984	photosystem I subunit PsaO	-1,27	434,08	0,15	8,98056E-16
k141_279621	photosystem I subunit PsaO	2,07	183,93	0,31	5,08598E-10
k141_364340	photosystem I subunit PsaO	-0,87	256,33	0,13	1,02666E-09
k141_177301	photosystem I subunit PsaO	1,99	456,74	0,31	2,19456E-09
k141_266794	photosystem I subunit PsaO	-1,42	64,67	0,25	1,38501E-07
k141_163809	photosystem I subunit PsaO	-1,56	297,28	0,39	0,000274167

Supplementary Table S5

ORF	Rank3	Log2Fold Change	base Mean	lfcSE	padj
k141_1476	photosystem I subunit X (PsaK, PsaK1)	2,70	240,69	0,26	6,20259E-23
k141_518292	photosystem I subunit X (PsaK, PsaK1)	-1,44	47,35	0,22	1,62293E-09
k141_312007	photosystem I subunit X (PsaK, PsaK1)	1,85	515,72	0,30	5,85316E-09
k141_537570	photosystem I subunit X (PsaK, PsaK1)	2,61	3,81	0,65	0,000216023
k141_339993	photosystem I subunit X (PsaK, PsaK1)	3,44	2,04	0,96	0,001236292
k141_455562	photosystem I subunit XI (PsaL)	-8,58	48,31	0,69	2,27354E-33
k141_190608	photosystem I subunit XI (PsaL)	2,51	488,98	0,27	2,95535E-19
k141_334187	photosystem I subunit XI (PsaL)	2,04	1026,47	0,28	1,09354E-11
k141_228666	photosystem I subunit XI (PsaL)	-1,43	57,81	0,24	2,35541E-08
k141_130801	photosystem I subunit XI (PsaL)	-2,68	7,10	0,57	1,6285E-05
k141_223209	photosystem I subunit XI (PsaL)	3,35	1,95	0,93	0,001124451
k141_489633	photosystem I subunit XI (PsaL)	-1,15	70,39	0,32	0,001262853
k141_268980	photosystem I subunit XI (PsaL)	2,96	3,67	0,86	0,001975307
k141_345504	Photosystem II 13 kDa protein Psb28 (similar to	2,07	49,94	0,28	4,9786E-12
k141_346335	Photosystem II 13 kDa protein Psb28 (similar to	1,91	149,55	0,27	1,56695E-11
k141_507137	Photosystem II 13 kDa protein Psb28 (similar to	3,84	17,85	0,86	4,37599E-05
k141_195871	Photosystem II 13 kDa protein Psb28 (similar to	4,05	3,03	0,94	8,19761E-05
k141_422781	Photosystem II 13 kDa protein Psb28 (similar to	4,56	2,10	1,10	0,000153749
k141_326733	Photosystem II 13 kDa protein Psb28 (similar to	0,98	68,92	0,25	0,000270002
k141_368506	Photosystem II 13 kDa protein Psb28 (similar to	-0,63	860,68	0,16	0,000457751
k141_29951	Photosystem II 13 kDa protein Psb28 (similar to	-1,61	8,90	0,47	0,001842017
k141_345096	Photosystem II 13 kDa protein Psb28 (similar to	2,10	3,56	0,67	0,00472691
k141_539900	Photosystem II manganese-stabilizing protein	-5,11	40,18	0,33	2,43983E-51
k141_557213	Photosystem II manganese-stabilizing protein	-1,51	76,40	0,17	4,08634E-18
k141_324219	Photosystem II manganese-stabilizing protein	2,18	188,96	0,25	1,28753E-16
k141_60116	Photosystem II manganese-stabilizing protein	-1,35	357,90	0,18	4,44854E-13
k141_564911	Photosystem II manganese-stabilizing protein	-5,32	3,59	0,84	2,43494E-09
k141_23099	Photosystem II manganese-stabilizing protein	4,02	4,10	0,85	1,34675E-05
k141_44560	Photosystem II manganese-stabilizing protein	-0,41	1752,70	0,11	0,001241491
k141_503320	Photosystem II manganese-stabilizing protein	-1,79	60,92	0,52	0,001907702
k141_520673	Photosystem II manganese-stabilizing protein	-3,14	2,20	1,08	0,009658933
k141_297088	Photosystem II oxygen evolving complex	2,93	975,71	0,24	1,4892E-31
k141_194023	Photosystem II oxygen evolving complex	-1,21	454,72	0,14	4,66468E-17
k141_176594	Photosystem II oxygen evolving complex	-1,90	99,95	0,26	7,00361E-12
k141_207737	Photosystem II oxygen evolving complex	2,07	4,28	0,67	0,005674152
k141_70397	Photosystem II oxygen evolving complex	2,84	5,46	0,94	0,006513891
k141_437270	Possible alpha/beta hydrolase superfamily,	-0,61	7465,76	0,09	3,82026E-10
k141_485748	Possible alpha/beta hydrolase superfamily,	1,12	50,58	0,23	6,39876E-06
k141_384269	Possible alpha/beta hydrolase superfamily,	-2,15	3,65	0,60	0,001212572
k141_89459	Possible alpha/beta hydrolase superfamily,	1,36	12,83	0,40	0,00231469
k141_361938	Possible alpha/beta hydrolase superfamily,	-2,35	2,40	0,75	0,004673889
k141_335555	Ribulose bisphosphate carboxylase small chain	3,84	1820,06	0,23	6,06385E-62
k141_412498	Ribulose bisphosphate carboxylase small chain	-1,96	375,58	0,13	1,31066E-46
k141_434083	Ribulose bisphosphate carboxylase small chain	-1,97	295,25	0,14	3,78121E-43
k141_28060	Ribulose bisphosphate carboxylase small chain	3,27	755,07	0,23	9,71614E-43
k141_22624	Ribulose bisphosphate carboxylase small chain	3,18	1592,28	0,25	9,94082E-34
k141_177671	Ribulose bisphosphate carboxylase small chain	2,93	1905,83	0,24	5,45526E-33
k141_323426	Ribulose bisphosphate carboxylase small chain	-1,71	168,68	0,15	5,82237E-29
k141_461167	Ribulose bisphosphate carboxylase small chain	-1,19	165,17	0,11	1,99631E-23
k141_317349	Ribulose bisphosphate carboxylase small chain	-6,94	11,07	0,68	7,29111E-23

Supplementary Table S5

ORF	Rank3	Log2Fold Change	base Mean	lfcSE	padj
**k141_35616**	**Ribulose bisphosphate carboxylase small chain**	2,59	369,15	0,26	5,17756E-22
**k141_117874**	**Ribulose bisphosphate carboxylase small chain**	2,24	121,05	0,22	7,8494E-22
**k141_276815**	**Ribulose bisphosphate carboxylase small chain**	2,49	608,45	0,27	1,18614E-18
**k141_27483**	**Ribulose bisphosphate carboxylase small chain**	2,51	12457,43	0,28	3,5801E-18
**k141_104258**	**Ribulose bisphosphate carboxylase small chain**	-6,32	19,29	0,71	1,46018E-17
**k141_268674**	**Ribulose bisphosphate carboxylase small chain**	2,49	1116,96	0,28	3,22833E-17
**k141_27789**	**Ribulose bisphosphate carboxylase small chain**	1,79	4879,06	0,21	1,79846E-16
**k141_50257**	**Ribulose bisphosphate carboxylase small chain**	3,81	11,57	0,46	6,52962E-15
**k141_436841**	**Ribulose bisphosphate carboxylase small chain**	2,66	29,06	0,35	3,77875E-13
**k141_162540**	**Ribulose bisphosphate carboxylase small chain**	-1,47	962,20	0,20	7,84327E-12
**k141_125305**	**Ribulose bisphosphate carboxylase small chain**	-1,23	326,57	0,17	8,52089E-12
**k141_75390**	**Ribulose bisphosphate carboxylase small chain**	1,76	261,90	0,25	7,07346E-11
**k141_498912**	**Ribulose bisphosphate carboxylase small chain**	4,44	10,52	0,65	1,26009E-10
**k141_394558**	**Ribulose bisphosphate carboxylase small chain**	1,72	440,22	0,25	1,6039E-10
**k141_267978**	**Ribulose bisphosphate carboxylase small chain**	2,04	1634,96	0,30	1,7359E-10
**k141_208364**	**Ribulose bisphosphate carboxylase small chain**	-0,79	1538,83	0,12	2,11571E-10
**k141_260136**	**Ribulose bisphosphate carboxylase small chain**	3,87	8,54	0,58	2,62989E-10
**k141_438106**	**Ribulose bisphosphate carboxylase small chain**	2,94	11,62	0,44	4,18627E-10
**k141_119881**	**Ribulose bisphosphate carboxylase small chain**	-1,07	739,46	0,17	1,0905E-09
**k141_357404**	**Ribulose bisphosphate carboxylase small chain**	-4,48	5,69	0,70	1,56366E-09
**k141_17347**	**Ribulose bisphosphate carboxylase small chain**	2,78	11,04	0,44	3,31128E-09
**k141_484488**	**Ribulose bisphosphate carboxylase small chain**	2,08	39,77	0,33	3,37734E-09
**k141_532535**	**Ribulose bisphosphate carboxylase small chain**	-3,81	18,23	0,61	4,70946E-09
**k141_97867**	**Ribulose bisphosphate carboxylase small chain**	-1,33	654,03	0,21	6,07163E-09
**k141_398759**	**Ribulose bisphosphate carboxylase small chain**	3,24	12,53	0,53	9,57611E-09
**k141_326291**	**Ribulose bisphosphate carboxylase small chain**	1,62	325,24	0,28	5,40512E-08
**k141_542033**	**Ribulose bisphosphate carboxylase small chain**	2,65	21,62	0,46	6,95043E-08
**k141_520986**	**Ribulose bisphosphate carboxylase small chain**	-3,92	3,90	0,74	9,10371E-07
**k141_564046**	**Ribulose bisphosphate carboxylase small chain**	2,66	8,09	0,51	1,13718E-06
**k141_548356**	**Ribulose bisphosphate carboxylase small chain**	-1,44	28,96	0,28	1,81944E-06
**k141_500438**	**Ribulose bisphosphate carboxylase small chain**	-0,76	1492,40	0,16	1,79749E-05
**k141_458236**	**Ribulose bisphosphate carboxylase small chain**	4,26	2,51	0,92	2,08526E-05
**k141_123668**	**Ribulose bisphosphate carboxylase small chain**	1,58	28,45	0,34	2,19669E-05
**k141_45870**	**Ribulose bisphosphate carboxylase small chain**	2,46	12,90	0,55	4,54645E-05
**k141_187455**	**Ribulose bisphosphate carboxylase small chain**	-0,92	44,82	0,21	6,42205E-05
**k141_44305**	**Ribulose bisphosphate carboxylase small chain**	-0,64	5918,56	0,15	6,70447E-05
**k141_508172**	**Ribulose bisphosphate carboxylase small chain**	-1,40	17,19	0,35	0,000308423
**k141_420712**	**Ribulose bisphosphate carboxylase small chain**	0,49	507,38	0,13	0,000351997
**k141_288531**	**Ribulose bisphosphate carboxylase small chain**	7,16	12,76	1,95	0,000867456
**k141_180320**	**Ribulose bisphosphate carboxylase small chain**	0,45	562,35	0,12	0,00106172
**k141_56135**	**Ribulose bisphosphate carboxylase small chain**	1,34	11,35	0,37	0,001121168
**k141_53869**	**Ribulose bisphosphate carboxylase small chain**	0,85	4119,67	0,26	0,002775729
**k141_283875**	**Ribulose bisphosphate carboxylase small chain**	0,39	1820,75	0,12	0,002785151
**k141_377448**	**Ribulose bisphosphate carboxylase small chain**	-1,24	404,83	0,37	0,002802405
**k141_186696**	**Ribulose bisphosphate carboxylase small chain**	3,63	2,30	1,12	0,003505223
**k141_373016**	**Ribulose bisphosphate carboxylase small chain**	0,39	1100,02	0,12	0,00355818
**k141_339217**	**Ribulose bisphosphate carboxylase small chain**	-1,95	15,19	0,60	0,003593552
**k141_291576**	**Ribulose bisphosphate carboxylase small chain**	0,38	1946,95	0,12	0,00399674
**k141_418272**	**Ribulose bisphosphate carboxylase small chain**	0,33	8122,41	0,10	0,004077269
**k141_501798**	**Ribulose bisphosphate carboxylase small chain**	0,32	3247,47	0,11	0,006033711

Supplementary Table S5

ORF	Rank3	Log2Fold Change	base Mean	lfcSE	padj
**k141_193554**	**Ribulose bisphosphate carboxylase small chain**	0,61	421,72	0,20	0,006589074
**k141_399059**	**S-formylglutathione hydrolase (EC 3.1.2.12)**	-6,50	8,19	0,71	1,82928E-18
**k141_114710**	**S-formylglutathione hydrolase (EC 3.1.2.12)**	2,28	21,22	0,41	2,27241E-07
**k141_399265**	**S-formylglutathione hydrolase (EC 3.1.2.12)**	-5,31	3,57	0,97	3,20614E-07
**k141_144658**	**S-formylglutathione hydrolase (EC 3.1.2.12)**	-0,46	1151,64	0,12	0,000295845
**k141_549493**	**S-formylglutathione hydrolase (EC 3.1.2.12)**	2,58	5,22	0,67	0,000507979
**k141_541479**	**S-formylglutathione hydrolase (EC 3.1.2.12)**	3,97	2,86	1,20	0,002839428
**k141_422938**	**Sucrose-6-phosphate hydrolase (EC 3.2.1.B3)**	-5,19	23,69	0,56	1,42418E-18
**k141_390600**	**Sucrose-6-phosphate hydrolase (EC 3.2.1.B3)**	3,50	22,42	0,44	3,03372E-14
**k141_17690**	**Sucrose-6-phosphate hydrolase (EC 3.2.1.B3)**	3,09	26,00	0,41	4,92529E-13
**k141_330797**	**Sucrose-6-phosphate hydrolase (EC 3.2.1.B3)**	-1,31	358,84	0,19	4,44635E-11
**k141_83755**	**Sucrose-6-phosphate hydrolase (EC 3.2.1.B3)**	4,79	5,84	0,87	2,65083E-07
**k141_556054**	**Sucrose-6-phosphate hydrolase (EC 3.2.1.B3)**	2,65	9,73	0,53	3,10383E-06
**k141_121216**	**Sucrose-6-phosphate hydrolase (EC 3.2.1.B3)**	2,88	4,97	0,68	0,000104656
**k141_128619**	**Sucrose-6-phosphate hydrolase (EC 3.2.1.B3)**	4,59	3,05	1,12	0,000171844
**k141_525775**	**Uridine diphosphate glucose pyrophosphatase**	1,58	16,23	0,42	0,0005962
