# Supplementary material for: Temperature Stress Induces Shift From Co-Existence to Competition for Organic Carbon in Microalgae-Bacterial Photobioreactor Community – Enabling Continuous Production of Microalgal Biomass
Source: Front Microbiol. 2021 Feb 11;12:607601. doi: 10.3389/fmicb.2021.607601 (PMC7905023; doi:10.3389/fmicb.2021.607601)
Supplement: Supplementary file 4 [file Data_Sheet_4.pdf]

Supplementary Table S6

ORF	Rank3	log2Fold Change	base Mean	lfcSE	padj
k141_31579	Aconitate hydratase (EC 4.2.1.3)	-5,98	5,59	1,58	0,000774443
k141_13947	Aconitate hydratase (EC 4.2.1.3)	1,33	8,11	0,50	0,021954354
k141_54246	Alkaline phosphatase (EC 3.1.3.1)	3,79	4,10	0,92	0,000223609
k141_37394	Alkaline phosphatase (EC 3.1.3.1)	-4,02	2,16	1,14	0,001921464
k141_59592	Alkaline phosphatase (EC 3.1.3.1)	3,09	3,99	0,93	0,003762151
k141_17473	Branched-chain amino acid transport system permease protein LivM (TC 3.A.1.4.1)	-4,07	1,53	1,11	0,00114315
k141_24322	Branched-chain amino acid transport system permease protein LivM (TC 3.A.1.4.1)	2,82	1,40	1,03	0,018628112
k141_51868	Branched-chain amino acid transport system permease protein LivM (TC 3.A.1.4.1)	3,11	2,13	1,19	0,024481397
k141_43221	Cytochrome c oxidase polypeptide II (EC 1.9.3.1)	-2,17	1056,79	0,17	2,85186E-36
k141_7224	Cytochrome c oxidase polypeptide II (EC 1.9.3.1)	-3,94	5,28	0,68	9,29729E-08
k141_23261	Cytochrome c oxidase polypeptide II (EC 1.9.3.1)	-4,54	2,08	0,98	2,42565E-05
k141_47814	Cytochrome c oxidase polypeptide II (EC 1.9.3.1)	-0,88	117,41	0,20	0,00012113
k141_59898	Cytochrome c oxidase polypeptide II (EC 1.9.3.1)	-0,61	26320,03	0,15	0,000245374
k141_35595	D-allose ABC transporter, substrate-binding component	3,15	4,07	0,74	0,000130365
k141_47188	Dihydrolipoamide dehydrogenase of pyruvate dehydrogenase complex (EC 1.8.1.4)	3,36	0,93	1,35	0,033097056
k141_16690	Enolase (EC 4.2.1.11)	3,25	9,51	0,61	1,086E-06
k141_43148	Enolase (EC 4.2.1.11)	2,40	4,53	0,67	0,001482474
k141_22855	Fructose ABC transporter, permease component FrcC	3,85	1,88	1,37	0,015204698
k141_33958	Fructose ABC transporter, substrate-binding component	2,60	2,20	0,98	0,022553315
k141_12848	Fructose-bisphosphate aldolase class I (EC 4.1.2.13)	-5,39	5,41	0,77	5,22222E-11
k141_49365	Fructose-bisphosphate aldolase class I (EC 4.1.2.13)	3,50	3,67	0,82	0,00012055
k141_58933	Fructose-bisphosphate aldolase class I (EC 4.1.2.13)	4,20	1,67	1,06	0,000396658
k141_55618	Fructose-bisphosphate aldolase class I (EC 4.1.2.13)	3,13	1,59	0,99	0,005532763
k141_11320	Fructose-bisphosphate aldolase class I (EC 4.1.2.13)	-4,22	1,68	1,63	0,026155986
k141_39431	Fructose-bisphosphate aldolase class I (EC 4.1.2.13)	-1,43	6,12	0,57	0,032660249
k141_12469	Fructose-bisphosphate aldolase class I (EC 4.1.2.13)	2,81	1,46	1,14	0,034485957
k141_36738	Glucose-6-phosphate isomerase (EC 5.3.1.9)	2,46	3,44	0,77	0,005213382
k141_37676	Glycerol-3-phosphate ABC transporter, permease protein UgpA (TC 3.A.1.1.3)	3,69	6,02	0,72	2,88552E-06
k141_56999	Glycerol-3-phosphate ABC transporter, permease protein UgpA (TC 3.A.1.1.3)	-3,26	1,79	0,93	0,001905746
k141_50635	Glycerol-3-phosphate ABC transporter, permease protein UgpA (TC 3.A.1.1.3)	0,97	13,54	0,37	0,023351162
k141_10947	Glycerol-3-phosphate ABC transporter, permease protein UgpA (TC 3.A.1.1.3)	3,23	0,86	1,37	0,044085231
k141_29626	High-affinity leucine-specific transport system, periplasmic binding protein LivK (TC 3.A.1.4.1)	3,13	18,02	0,44	3,22239E-11
k141_39250	High-affinity leucine-specific transport system, periplasmic binding protein LivK (TC 3.A.1.4.1)	3,10	1,21	1,24	0,031535101
k141_59432	Hydroxymethylpyrimidine ABC transporter, transmembrane component	4,42	1,94	0,98	4,27226E-05
k141_45720	Inositol transport system sugar-binding protein	2,15	39,27	0,30	3,09932E-11
k141_54705	Isocitrate dehydrogenase [NADP] (EC 1.1.1.42)	3,61	2,37	1,03	0,002026596
k141_21253	Isocitrate dehydrogenase [NADP] (EC 1.1.1.42)	-4,60	2,15	1,40	0,004074065
k141_50640	Isocitrate dehydrogenase [NADP] (EC 1.1.1.42)	-0,67	47,34	0,24	0,015126757
k141_20843	L-proline glycine betaine binding ABC transporter protein ProX (TC 3.A.1.12.1)	3,49	7,62	0,69	4,70509E-06
k141_56658	L-proline glycine betaine binding ABC transporter protein ProX (TC 3.A.1.12.1)	-2,87	3,41	0,83	0,002191144
k141_59196	L-proline glycine betaine binding ABC transporter protein ProX (TC 3.A.1.12.1)	-3,35	0,93	1,38	0,037445065

Supplementary Table S6

ORF	Rank3	log2Fold Change	base Mean	lfcSE	padj
k141_51802	Lipopolysaccharide ABC transporter, ATP-binding protein	2,44	6,03	0,59	0,000214798
k141_32991	Maltose/maltodextrin ABC transporter, substrate binding periplasmic protein MalE	-3,87	1,29	1,05	0,001139841
k141_47633	Maltose/maltodextrin ABC transporter, substrate binding periplasmic protein MalE	4,27	1,77	1,43	0,009500684
k141_57294	Maltose/maltodextrin ABC transporter, substrate binding periplasmic protein MalE	1,85	8,45	0,67	0,017164573
k141_8924	Phosphoglycerate kinase (EC 2.7.2.3)	3,36	26,45	0,43	1,78296E-13
k141_38735	Phosphoglycerate kinase (EC 2.7.2.3)	2,50	60,85	0,36	7,48034E-11
k141_3813	Phosphoglycerate kinase (EC 2.7.2.3)	3,91	2,80	1,00	0,000466879
k141_49305	Phosphoglycerate kinase (EC 2.7.2.3)	2,74	2,28	0,88	0,006406882
k141_24002	Phosphoglycerate kinase (EC 2.7.2.3)	3,04	1,74	1,03	0,010107738
k141_57902	Phosphoglycerate kinase (EC 2.7.2.3)	3,78	1,25	1,29	0,011010229
k141_29530	Phosphoglycerate kinase (EC 2.7.2.3)	-3,98	1,41	1,60	0,033247984
k141_456	Predicted maltose-specific TonB-dependent receptor	3,15	8,25	0,62	3,66062E-06
k141_39854	Predicted maltose-specific TonB-dependent receptor	2,96	2,36	1,00	0,010635674
k141_46513	Predicted maltose-specific TonB-dependent receptor	3,10	1,40	1,20	0,027148828
k141_30491	Predicted nucleoside ABC transporter, substrate-binding component	3,75	11,01	0,57	8,029E-10
k141_39869	Predicted nucleoside ABC transporter, substrate-binding component	3,04	4,61	0,83	0,001226505
k141_7925	Predicted nucleoside ABC transporter, substrate-binding component	-3,41	2,05	1,03	0,003838208
k141_7594	Predicted nucleoside ABC transporter, substrate-binding component	-3,00	1,05	1,10	0,018878807
k141_58236	Putative ubiquinol-cytochrome c reductase complex core protein II (subunit COR2)	-0,83	61,57	0,27	0,006701191
k141_48276	Pyrimidine ABC transporter, substrate-binding component	1,96	4,13	0,57	0,002365419
k141_47831	Pyrimidine ABC transporter, substrate-binding component	2,51	2,90	0,81	0,007193879
k141_9919	Pyruvate dehydrogenase E1 component beta subunit (EC	4,08	3,32	0,94	8,84945E-05
k141_11694	Pyruvate dehydrogenase E1 component beta subunit (EC	2,50	4,65	0,59	0,000140436
k141_63047	Pyruvate dehydrogenase E1 component beta subunit (EC	-3,84	1,26	1,44	0,021203965
k141_7151	Pyruvate dehydrogenase E1 component beta subunit (EC	3,88	1,35	1,47	0,023422409
k141_59052	Pyruvate dehydrogenase E1 component beta subunit (EC	-3,83	1,25	1,60	0,041089194
k141_13871	Ribulose bisphosphate carboxylase large chain (EC 4.1.1.39)	2,17	2289,85	0,26	3,39565E-15
k141_37859	Ribulose bisphosphate carboxylase large chain (EC 4.1.1.39)	6,09	6,15	0,75	1,9968E-14
k141_55744	Ribulose bisphosphate carboxylase large chain (EC 4.1.1.39)	3,11	240,54	0,51	1,57464E-08
k141_20496	Ribulose bisphosphate carboxylase large chain (EC 4.1.1.39)	-3,06	5,81	0,60	3,144E-06
k141_16010	Ribulose bisphosphate carboxylase large chain (EC 4.1.1.39)	-1,12	27,44	0,24	2,70365E-05
k141_2223	Ribulose bisphosphate carboxylase large chain (EC 4.1.1.39)	3,61	8,63	0,79	3,16264E-05
k141_4662	Ribulose bisphosphate carboxylase large chain (EC 4.1.1.39)	-1,26	22,81	0,31	0,000328377
k141_21520	Ribulose bisphosphate carboxylase large chain (EC 4.1.1.39)	-1,18	17,54	0,35	0,00289039
k141_8226	Ribulose bisphosphate carboxylase large chain (EC 4.1.1.39)	-0,83	26,14	0,27	0,00717919
k141_8005	Ribulose bisphosphate carboxylase large chain (EC 4.1.1.39)	-1,40	8,77	0,51	0,018207074
k141_7229	Ribulose bisphosphate carboxylase large chain (EC 4.1.1.39)	-1,01	7,34	0,38	0,024349151
k141_8452	Ribulose bisphosphate carboxylase large chain (EC 4.1.1.39)	2,75	1,44	1,10	0,031699146
k141_11889	Ribulose bisphosphate carboxylase small chain (EC 4.1.1.39)	2,29	36,60	0,24	2,29285E-19
k141_30689	Ribulose bisphosphate carboxylase small chain (EC 4.1.1.39)	2,49	602,21	0,27	2,71891E-18
k141_24330	Ribulose bisphosphate carboxylase small chain (EC 4.1.1.39)	2,65	55,37	0,31	2,64762E-16
k141_35273	Ribulose bisphosphate carboxylase small chain (EC 4.1.1.39)	2,44	30,05	0,39	6,26768E-09
k141_5705	Ribulose bisphosphate carboxylase small chain (EC 4.1.1.39)	3,97	4,14	0,75	1,06974E-06
k141_49747	Ribulose bisphosphate carboxylase small chain (EC 4.1.1.39)	3,00	10,09	0,60	4,81705E-06
k141_29998	Ribulose bisphosphate carboxylase small chain (EC 4.1.1.39)	1,32	64,98	0,34	0,000449862
k141_22912	Ribulose bisphosphate carboxylase small chain (EC 4.1.1.39)	0,55	3418,09	0,18	0,008454552
k141_44503	Ribulose bisphosphate carboxylase small chain (EC 4.1.1.39)	3,59	1,10	1,33	0,019815877

Supplementary Table S6

ORF	Rank3	log2Fold Change	base Mean	lfcSE	padj
**k141_28925**	**Ribulose bisphosphate carboxylase small chain (EC 4.1.1.39)**	-0,94	24,41	0,39	0,039313569
**k141_21798**	**Succinate dehydrogenase flavoprotein subunit (EC 1.3.99.1)**	-5,10	4,35	1,20	0,000142862
**k141_59421**	**Succinate dehydrogenase flavoprotein subunit (EC 1.3.99.1)**	3,19	1,90	1,00	0,005128273
**k141_37177**	**Succinate dehydrogenase flavoprotein subunit (EC 1.3.99.1)**	-3,55	1,03	1,33	0,022145638
**k141_31966**	**Succinate dehydrogenase flavoprotein subunit (EC 1.3.99.1)**	-3,59	1,10	1,49	0,039903043
**k141_11338**	**Survival protein SurA precursor (Peptidyl-prolyl cis-trans isomerase SurA) (EC 5.2.1.8)**	3,27	1,82	1,11	0,010425095
**k141_9067**	**TonB system biopolymer transport component**	2,21	15,42	0,54	0,00027552
**k141_22915**	**TRAP-type transport system, small permease component, predicted N-acetylneuraminate transporter**	1,87	14,06	0,47	0,000398099
**k141_57849**	**ubiquinol cytochrome C oxidoreductase, cytochrome C1**	-4,47	2,78	0,98	3,9872E-05
**k141_11289**	**UDP-glucose 4-epimerase (EC 5.1.3.2)**	3,03	690,67	0,17	9,19947E-65
**k141_13430**	**UDP-glucose 4-epimerase (EC 5.1.3.2)**	-1,32	7637,56	0,15	1,7183E-17
**k141_40417**	**UDP-glucose 4-epimerase (EC 5.1.3.2)**	-1,75	165,74	0,35	3,69952E-06
**k141_22419**	**UDP-glucose 4-epimerase (EC 5.1.3.2)**	-1,11	38,79	0,25	5,13247E-05
**k141_22409**	**UDP-glucose 4-epimerase (EC 5.1.3.2)**	-1,17	22,76	0,28	0,000163799
**k141_48317**	**UDP-glucose 4-epimerase (EC 5.1.3.2)**	2,71	3,30	0,76	0,001701538
**k141_40154**	**UDP-glucose 4-epimerase (EC 5.1.3.2)**	3,01	7,43	0,95	0,005679232
**k141_17569**	**UDP-glucose 4-epimerase (EC 5.1.3.2)**	1,35	6,09	0,48	0,01421208
**k141_23615**	**UDP-glucose 4-epimerase (EC 5.1.3.2)**	-2,80	1,40	1,00	0,016077701
**k141_47642**	**V-type proton ATPase V0 sector subunit a**	0,79	13,75	0,29	0,020643679
**k141_50871**	**Various polyols ABC transporter, periplasmic substrate-binding protein**	-3,67	11,58	0,60	1,15767E-08
**k141_28099**	**Various polyols ABC transporter, periplasmic substrate-binding protein**	2,19	9,06	0,53	0,000189676
**k141_7109**	**Various polyols ABC transporter, periplasmic substrate-binding protein**	-2,64	2,91	0,76	0,002307177
**k141_58284**	**Various polyols ABC transporter, periplasmic substrate-binding protein**	-3,43	1,42	0,99	0,002346934
**k141_34066**	**Various polyols ABC transporter, periplasmic substrate-binding protein**	2,59	1,72	1,06	0,03704447
**k141_60497**	**Various polyols ABC transporter, periplasmic substrate-binding protein**	-3,05	1,12	1,26	0,037532512
**k141_30628**	**Various polyols ABC transporter, periplasmic substrate-binding protein**	3,06	1,56	1,31	0,04679281
**k141_62180**	**Xylose ABC transporter, periplasmic xylose-binding protein**	-5,61	16,18	0,68	9,02739E-15
**k141_8378**	**Xylose ABC transporter, periplasmic xylose-binding protein**	1,83	10,74	0,40	3,92508E-05
**k141_9465**	**Xylose ABC transporter, periplasmic xylose-binding protein**	-3,35	1,35	1,11	0,009027317
**k141_62556**	**Xylose ABC transporter, periplasmic xylose-binding protein**	3,23	1,88	1,31	0,034688169
